# A Systematic Review of the Clinical Efficacy and Safety of CFTR Modulators in Cystic Fibrosis

**DOI:** 10.1038/s41598-019-43652-2

**Published:** 2019-05-10

**Authors:** Al-Rahim R. Habib, Majid Kajbafzadeh, Sameer Desai, Connie L. Yang, Kate Skolnik, Bradley S. Quon

**Affiliations:** 1 School of Medicine, The University of Sydney, Sydney, Australia; 2 School of Population and Public Health, University of British Columbia, Vancouver, Canada; 3 Division of Respiratory Medicine, Department of Pediatrics, University of British Columbia, Vancouver, Canada; 4 Division of Respirology, Department of Medicine, University of Calgary, Alberta, Canada; 5 Centre for Heart Lung Innovation, St. Paul’s Hospital, Department of Medicine, University of British Columbia, Vancouver, Canada

**Keywords:** Cystic fibrosis, Molecular medicine

## Abstract

Several placebo-controlled trials have been recently published evaluating novel therapies targeting the defective CFTR protein. This systematic review examines the clinical efficacy and safety of CFTR modulators in individuals with cystic fibrosis (CF) with specific genetic mutations. Online sources were searched for placebo-controlled, parallel-design clinical trials investigating CFTR modulators from January 1, 2005 to March 31, 2018. The primary outcome of interest was FEV_1_% predicted (ppFEV_1_). Fourteen RCTs met our eligibility criteria. The largest improvement in ppFEV_1_ favouring treatment was observed for ivacaftor (IVA) in G551D individuals (≥6 years old). Both tezacaftor-ivacaftor (TEZ-IVA) and lumacaftor-ivacaftor (LUM-IVA) also improved ppFEV_1_ in F508del homozygous individuals but there was increased reporting of respiratory adverse events with LUM-IVA compared to placebo. IVA also significantly improved ppFEV_1_ in a sub-group of individuals ≥18 years old with an R117H mutation. No significant improvements in ppFEV_1_ were observed for IVA, LUM, or TEZ in F508del homozygous individuals, LUM or LUM-IVA in F508del heterozygous individuals, or ataluren in individuals with a nonsense mutation. Significant improvements in ppFEV_1_ and other clinical outcomes were observed for IVA in G551D individuals, TEV-IVA and LUM-IVA in F508del homozygous individuals, and IVA in adults with a R117H mutation.

## Introduction

Cystic fibrosis (CF) is a genetic condition caused by dysfunction of the cystic fibrosis transmembrane conductance regulator (CFTR) protein. CFTR is located at the apical surface of epithelial cells and the absence of CFTR activity leads to loss of chloride secretion and deficient fluid transport^1^. This results in thick and sticky secretions involving a range of epithelial tissues such as the airways and pancreatic ducts, eventually culminating in end-organ damage and failure. Since the discovery of the CFTR gene in 1989^2^, significant progress has been made in the understanding of how CFTR gene mutations alter protein structure and function leading to reduced CFTR activity^3^.

Although over 2000 variants in the CFTR gene have been identified to date, F508del accounts for most CFTR alleles in patients with CF. This particular mutation leads to abnormal CFTR folding and trafficking causing reduced delivery of CFTR to the cell surface^4^. Another class of CFTR mutations, referred to as “nonsense” mutations, leads to a premature termination codon and reduced synthesis and hence delivery of CFTR to the cell surface^5^. In contrast, “gating” mutations are missense mutations that lead to CFTR proteins that are sufficiently synthesized, processed and trafficked to the cell surface but once they arrive they have defective channel opening leading to diminished chloride secretion^6^.

With advances in our understanding of CFTR biology, a new class of small molecule therapies, referred to as CFTR modulators, have been identified using high-throughput small molecule screening; these drugs are unique as they directly target molecular defects in the CFTR protein to increase CFTR activity^7–11^. For example, CFTR “potentiators” are small molecules capable of increasing the amount of time the CFTR channel is spent in the open position and thus targets CFTR mutations with defective “gating”^10^. CFTR “correctors” are small molecules that can target mutations such as F508del as they can improve CFTR trafficking or transport to the cell surface by stabilizing the 3D conformation of the protein, even if misfolded^11^. Other CFTR modulators, including CFTR “amplifiers” and “translational read-through” agents increase the amount of CFTR protein produced, the latter being specific to mutations leading to a premature termination codon^12,13^.

In recent years, several placebo-controlled clinical trials have been conducted investigating the efficacy and safety of CFTR modulators but the results have varied depending on the specific CF genotype and therapy under investigation^8^. The primary objective of this systematic review was to evaluate the impact of CFTR modulators on lung function and other clinically important outcomes including pulmonary exacerbations, hospitalizations, respiratory symptoms, nutritional status, and adverse events in individuals with CF.

## Methods

### Search strategy

Our search strategy was developed in accordance with PRISMA guidelines^14^. A systematic search of online databases using key phrases was conducted to identify randomized, placebo-controlled trials published from January 1, 2005 to March 31, 2018. Online databases searched included: MEDLINE, EMBASE, ACP Journal Club, Cochrane Central Register for Controlled Trials (CENTRAL), Cochrane Database of Systematic Reviews (CDSR), Cochrane Methodology Register (CMR), Database of Abstracts of Reviews of Effects (DARE), Health Technology Assessment (HTA), and NHS Economic Evaluation Database (NHSEED). For comprehensiveness, clinical trial registries such as the European Medicines Agency, U.S. National Institute of Health, and the World Health Organization records were accessed and screened. We used the following key phrases which were designed to maximize sensitivity for detecting therapeutic trials in CF: (“cystic fibrosis” OR “CFTR”) AND (“drug therapy” OR “clinical trial”).

### Selection criteria

The literature search and abstracts were reviewed for eligibility independently by two investigators (A.R.H and M.K.). Randomized controlled trials (RCTs) with a parallel design comparing CFTR modulators (e.g. potentiators, correctors, translational read-through agents) to placebo in patients with CF were included. Study inclusion/exclusion were summarized in a PRISMA flow diagram^14^. The level of agreement in the articles selected for full text review and then for inclusion in the review by the two investigators were reported and discrepancies were resolved by the principal investigator (B.S.Q).

### Data extraction

The review protocol used in this study is available in the Appendix and was developed in accordance with the PRISMA statement^14^. Two reviewers (A.R.H. and M.K.) independently extracted data. The level of agreement in the data extracted broken down by study characteristics, risk of bias, and effects of the intervention by the two investigators were reported and discrepancies were resolved by the principal investigator (B.S.Q).

### Risk of bias assessment

Risk of bias was assessed using the Cochrane Risk of Bias tool^15^. A detailed review of the randomization process, blinding, and allocation sequence concealment was performed.

### Outcomes

Change in percent-predicted forced expiratory volume in one second (ppFEV_1_) was our primary outcome. Secondary efficacy outcomes included *protocol-defined* pulmonary exacerbations (PEx), hospitalization due to PEx, respiratory symptoms (*i*.*e*., Cystic Fibrosis Questionnaire-Revised (CFQ-R) Respiratory domain), and nutritional status (*i*.*e*., body mass index and weight). Adverse events with a prevalence of >10% (and involving >2 subjects) from either experimental or control groups, serious adverse events (including deaths) leading to treatment discontinuation, and the prevalence of elevated liver function tests (LFTs) were evaluated.

### Statistical analysis

The statistical analysis was performed using ReviewManager (RevMan 5.3, Copenhagen: The Nordic Cochrane Centre, The Cochrane Collaboration, 2014) in accordance with the Cochrane Handbook^16^. For each clinical outcome, the results were stratified by genotype and type/dose of CFTR modulator. If two or more studies evaluated the same drug at the same dose in the same genotype, the data was pooled using a fixed-effect meta-analysis (Appendix). For the primary outcome, ppFEV_1_, sub-group analyses were planned based on age and baseline ppFEV_1_.

## Results

### Study selection

**Figure 1:**
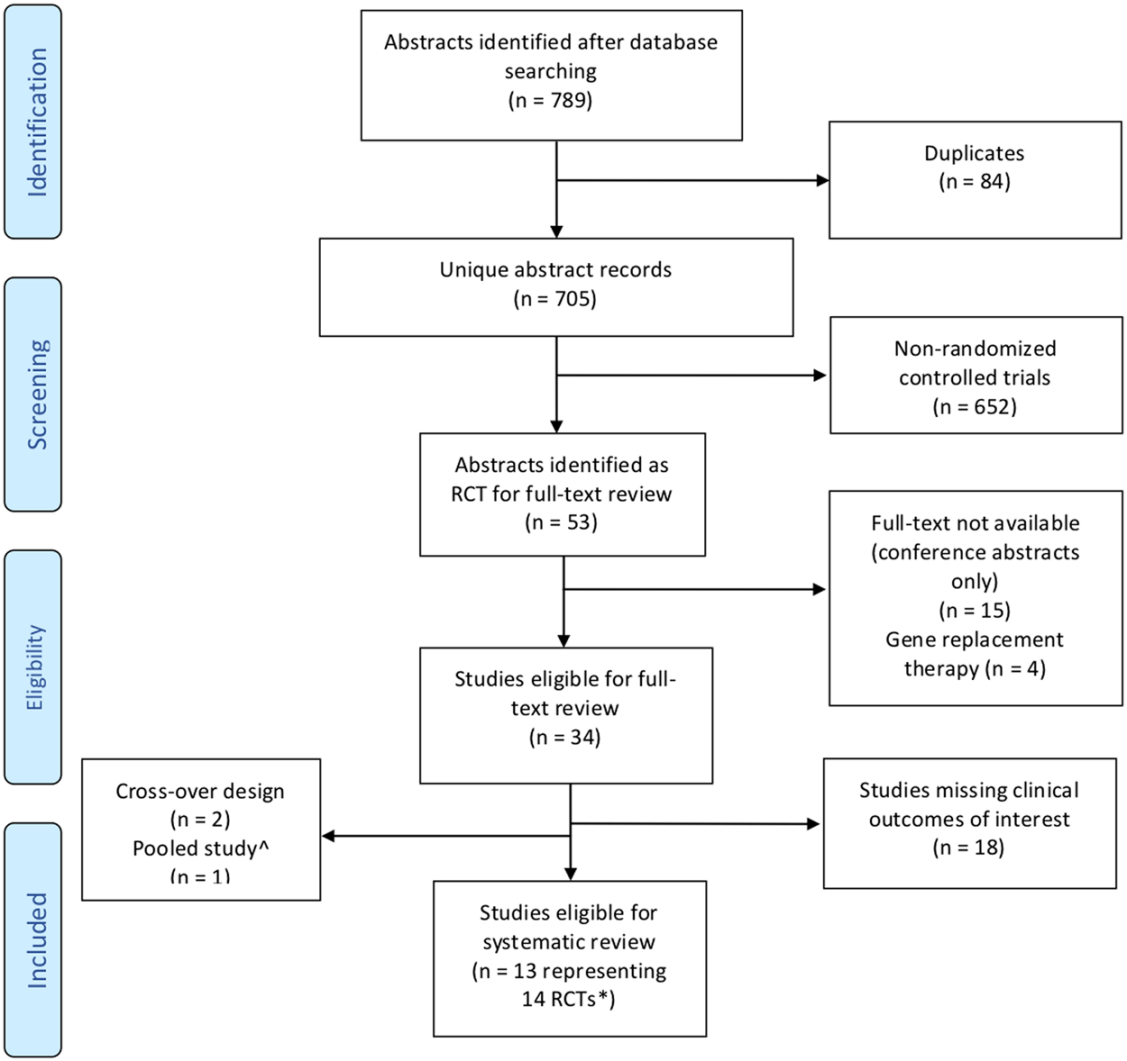
PRISMA Study Flow Diagram^42^. ^Subgroup analysis of a pooled study from TRAFFIC and TRANSPORT^43^. *One study by Wainwright *et al*.^17^ pooled data from two phase 3 RCTs (TRAFFIC and TRANSPORT) with identical study designs and methods of data analysis resulting in a total of 14 RCTs.

The search yielded a total of 789 potentially relevant articles and abstracts. Following full-text review, thirteen articles (14 placebo-controlled, parallel-group studies) met the inclusion and exclusion criteria (Fig. 1).

### Characteristics of included studies

A total of eight phase 3 and six phase 2 studies from thirteen original articles were identified. The article by Wainwright *et al*. included two phase 3 studies accounting for the discordance between the number of articles and studies^17^. The proposed class/mechanism of action for each CFTR modulator along with the number of studies evaluating the therapy is described in Table 1. Characteristics of the included studies and its participants are detailed in Table 2 and Appendix Table 1. The *a priori* outcomes of interest for the included studies are summarized in Appendix Table 2.

**Table 1:** CFTR Modulators Investigated in Phase 2 and 3 Clinical Trials.

Generic name	Genotypes investigated	Type of CFTR Modulator	No. of Studies
Ataluren	Nonsense mutation ≥ 1 allele	Translational readthrough agent – promotes ribosomal readthrough of premature termination codons to enable the production of full-length, functional CFTR	1
Ivacaftor (IVA)	F508del homozygous; F508del heterozygous G551D ≥ 1 allele; R117H ≥ 1 allele	CFTR “potentiator” – increases CFTR channel open probability (i.e., the fraction of time that the channel remains open)	5
Lumacaftor (LUM)	F508del homozygous	CFTR “corrector” – corrects CFTR misprocessing to increase the amount of cell surface-localized protein	2
Lumacaftor-ivacaftor (LUM-IVA)	F508del homozygous; F508del heterozygous	Combination CFTR corrector and potentiator	5
Tezacaftor (TEZ)	F508del homozygous	CFTR “corrector” – corrects CFTR misprocessing to increase the amount of cell surface-localized protein	1
Tezacaftor-ivacaftor (TEZ-IVA)	F508del homozygous; F508del/G551D	Combination CFTR corrector and potentiator	2

Abbreviations: CFTR = cystic fibrosis transmembrane conductance regulator.

**Table 2:** Characteristics of Phase 2 and 3 Clinical Trials Included in the Systematic Review.

Genotype	First Author and Year	Patient Characteristics							Intervention
		Phase	Countries	Treatment duration, wks	N	Sweat chloride, mmol/L Mean or Median (SD or range)	Age, yrs Mean or Median (SD or range)	ppFEV_1_ Mean or Median (SD or range)	Dose, Route, Frequency, and Duration
F508del homozygous	Flume (2012)^25^	2	USA	16	140	Mean 102 (80–136)	Mean 24 (12–52)	Mean 78 (40–129)	Ivacaftor: 150 mg PO BID
	Clancy (2012)^41^	2	Belgium, Canada, Germany, Netherlands, USA	4	89	Median 104 (66–129)	Median 26 (18–54)	Median 71 (34–128)	Lumacaftor: 25 mg PO daily 50 mg PO daily 100 mg PO daily 200 mg PO daily
	Boyle (2014) – Cohorts 1, 2, 3^24^	2	Australia, Belgium, France, Germany, New Zealand, UK, USA	Cohort 1: 3 wks Cohorts 2/3: 8 wks	186 Cohort 1: 62 Cohort 2: 109 Cohort 3: 15	Homozygous: Mean 100 (SD 8)	Mean 29 (SD 10)	Mean 67 (33–117)	Cohort 1: 1: Lumacaftor 200 mg PO OD monotherapy × 14d + Ivacaftor 150 mg PO BID combo × 7d 2: Lumacaftor 200 mg PO OD monotherapy × 14d + Ivacaftor 250 mg PO BID combo × 7d Cohort 2: 1: Lumacaftor 200 mg PO OD monotherapy × 28d + Ivacaftor 250 mg PO BID combo × 28d – homo only 2: Lumacaftor 400 mg PO OD monotherapy × 28d + Ivacaftor 250 mg PO BID combo × 28d – homo only 3: Lumacaftor 600 mg PO OD × 28d + Ivacaftor 250 mg PO BID combo × 28d – homo and hetero Cohort 3: 1: Lumacaftor 400 mg PO BID monotherapy × 28d + Ivacaftor 250 mg PO BID combo × 28d
	Wainwright (2015)^17^ TRAFFIC and TRANSPORT	3	Australia, Canada, Czech Republic, France, Germany, Ireland, Italy, Netherlands, Sweden, UK, USA	24	1108	N/A	Mean 25 (12–64)	Mean 60 (31–100)	Lumacaftor 600 mg PO OD + Ivacaftor 250 mg PO BID Lumacaftor 400 mg PO BID + Ivacaftor 250 mg PO BID
	Ratjen (2017)^19^	3	Australia, Belgium, Canada, Denmark, France, Germany, Sweden, UK, USA	24	204	Mean 103 (SD 10)	Mean 9 (SD 2)	Mean 90 (SD 12)	Lumacaftor 200 mg PO BID + Ivacaftor 250 mg PO BID
	Donaldson (2018)^22^	2	Canada, Germany, UK USA	4	172	Mean 99 (SD 12)	Mean 30 (SD 8)	Mean 60 (SD 14)	Dose escalation: 1: Tezacaftor 10 mg PO OD × 28d 2: Tezacaftor 30 mg PO OD × 28d 3: Tezacaftor 100 mg PO OD × 28d 4: Tezacaftor 150 mg PO OD × 28d 5: Tezacaftor 10 mg PO OD × 28d + Ivacaftor 150 mg PO BID 6: Tezacaftor 30 mg PO OD × 28d + Ivacaftor 150 mg PO BID 7: Tezacaftor 100 mg PO OD × 28d + Ivacaftor 150 mg PO BID 8: Tezacaftor 150 mg PO OD × 28d + Ivacaftor 150 mg PO BID Dose regimen testing: 1: Tezacaftor 100 mg PO OD + Ivacaftor 150 mg PO BID × 28d 2: Tezacaftor 100 mg PO OD + Ivacaftor 50 mg PO BID × 28d 3: Lumacaftor 50 mg PO BID × 28d + Ivacaftor 150 mg PO BID × 28d
	Taylor-Cousar (2017)^23^	3	Belgium, Canada, Denmark, France, Germany, Ireland, Italy, Netherlands, Spain, Sweden, Switzerland, UK, USA	24	504	Mean 100 (SD 10)	Mean 26 (SD 10)	Mean 60 (SD 15)	Tezacaftor 100 mg PO OD + Ivacaftor 150 mg PO BID
F508del heterozygous	Boyle (2014) – Cohort 2 only*^24^	2	Australia, Belgium, France, Germany, New Zealand, UK, USA	Cohort 2: 8 wks	Cohort 2: 109	Heterozygous: Mean 98 (SD 9)	Mean 29 (SD 10)	Mean 67 (33–117)	Cohort 2: Lumacaftor 600 mg PO OD × 28d + Ivacaftor 250 mg PO BID combo × 28d – homo and hetero
	Rowe (2017)^26^	2	Australia, Belgium, France, Germany, New Zealand, United Kingdom, USA	8	125	Mean 102 (SD 11)	Mean 30 (18–58)	Mean 62 (SD 14)	Lumacaftor 400 mg PO BID + Ivacaftor 250 mg PO BID
F508del/G551D	Donaldson (2018)^22^	2	Canada, Germany, UK USA	4	18	Mean 99 (SD 12)	Mean 30 (SD 8)	Mean 60 (SD 14)	Tezacaftor 100 mg PO OD + Ivacaftor 150 mg PO BID × 28d
G551D ≥ 1 allele	Accurso (2010)^28^	2	Canada, Germany, USA	4	19 (Part 2)	Median 96 (85–116)	Median 21 (18–42)	Median 69 (40–122)	Ivacaftor: 150 mg PO BID 250 mg PO BID
	Ramsey (2011)^20^	3	Australia, Canada, Czech Republic, France, Germany, Ireland, UK, USA	48	161	Mean 100 (58–128)	Mean 26 (12–53)	Mean 64 (32–98)	Ivacaftor: 150 mg PO BID
	Davies (2013)^21^	3	Australia, Canada, France, Germany, Ireland, UK, USA	48	52	Mean 105 (92–121)	Mean 9 (6–12)	Mean 84 (44–134)	Ivacaftor: 150 mg PO BID
R117H ≥ 1 allele	Moss (2015)^27^	3	Belgium, France, UK, USA	24	69	Mean 70 (SD 22)	Mean 31 (SD 17)	Mean 73 (SD 19)	Ivacaftor: 150 mg PO BID
Nonsense mutation ≥ 1 allele	Kerem (2014)^18^	3	Belgium, Canada, France, Germany, Israel, Italy, Netherlands, Spain, Sweden, UK, USA	48	238	Mean 98 (22–128)	Mean 23 (6–53)	Mean 61 (36–93)	Ataluren: 40 mg PO TID (10 mg/kg morning, 10 mg/kg midday, 20 mg/kg evening)

^*^Note: 2^nd^ allele had a mutation predicted to result in the lack of CFTR production or otherwise expected to be unresponsive to ivacaftor (based on *in vitro* testing).

Abbreviations: bid = twice daily; BMI = body mass index; CFQ-R = Cystic Fibrosis Questionnaire Revised; CFTR = cystic fibrosis transmembrane conductance regulator; FVC = forced vital capacity; IV = intravenous; LCI_2.5_ = lung clearance index or N_2_ washout until 2.5% of the starting N_2_ end-tidal concentration; N/A = not available; ppFEV_1_ = forced expiratory volume in 1 second (FEV_1_)% predicted; OD = once daily; PEx = pulmonary exacerbation; sd = standard deviation.

### Risk of bias of included studies

**Figure 2:**
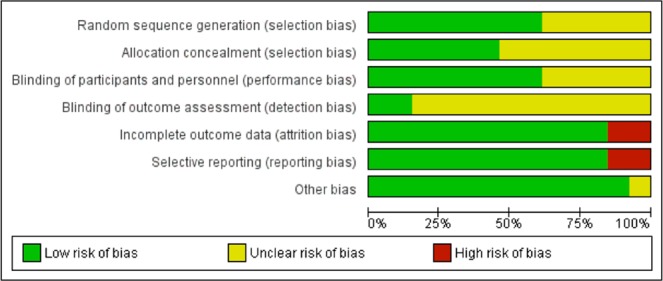
Risk of Bias Summary for Included Studies. Selective outcome reporting was noted for Kerem *et al*.^18^ as the study authors did not report in their full text publication all outcomes listed in their study protocol including antibiotic use and hospitalization due to CF-related symptoms, disruption to school or work due to CF-related symptoms, and pharmacokinetics. Similarly, Ramsey *et al*.^20^ did not report on all CFQ-R domain items or tertiary outcomes pre-defined in their clinical trial protocol including EQ-5D, oxygen saturation, and outpatient sick visits to the clinic or hospital for CF-related complications. Ratjen *et al*.^19^ did not report data on exacerbations (time to first, number) and the Treatment Satisfaction Questionnaire despite these being listed as secondary endpoints in the publication. Wainwright *et al*.^17^ did not report data on the EQ-5D or Treatment Satisfaction Questionnaire despite it being listed in their trial protocol.

Risk of bias for each included article is summarized in Appendix Fig. 1. Most studies were considered ‘low risk’ for selection, performance, and attrition bias (Fig. 2)^17–20^.

### Effects of the intervention

#### Primary outcome

**Figure 3:**
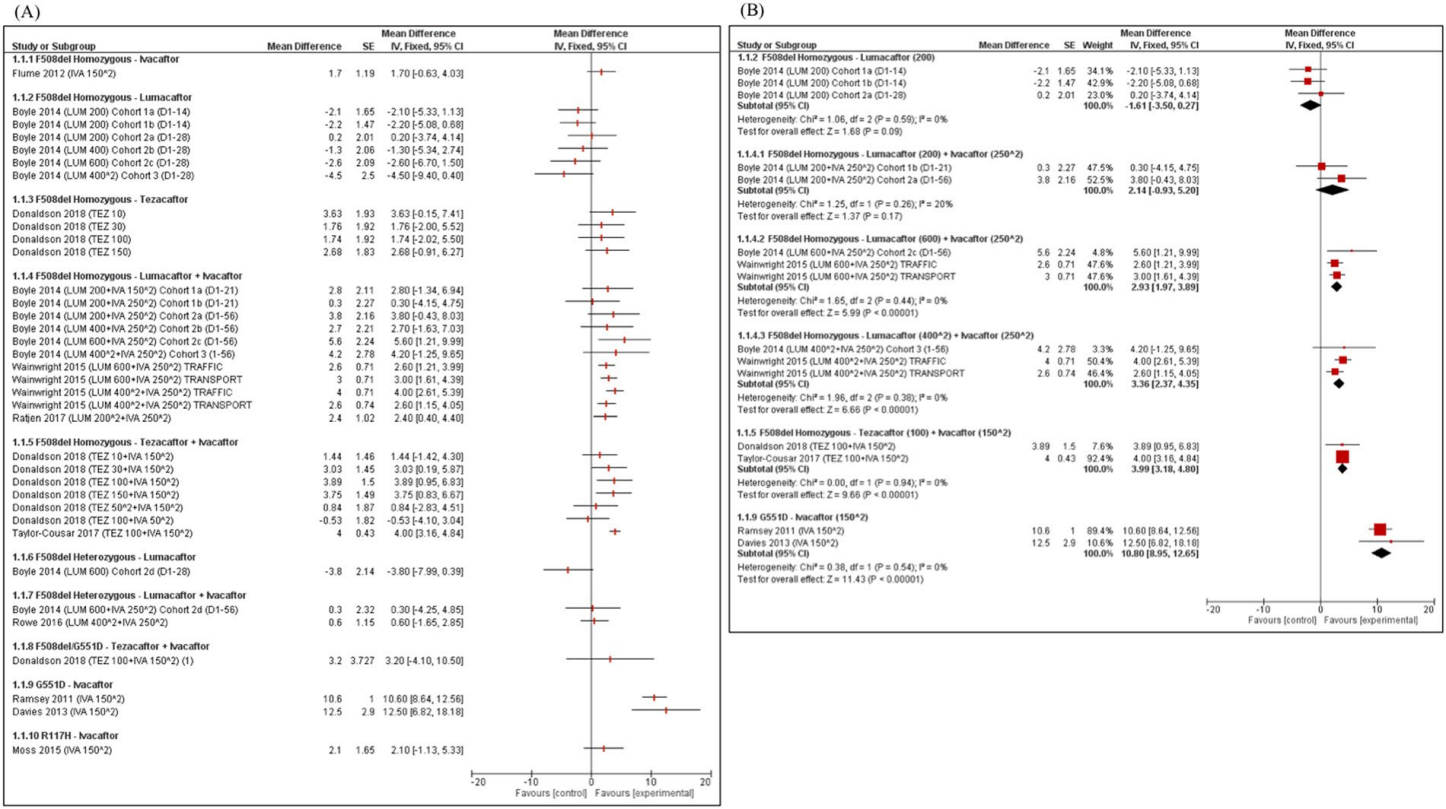
Absolute Difference in ppFEV_1_ for Patients Randomized to CFTR Modulators vs. Placebo. (**A**) Data from individual studies; (**B**) Meta-analysis combining data if identical CFTR modulator and dose. Footnote: (1) Individuals received IVA at baseline as part of routine clinical care and therefore the control group received IVA + Placebo. Abbreviations: D1–14 = day 1 to day 14; D1–21 = day 1 to day 21; D1–28 = day 1 to day 28; D1–56 = day 1 to day 56; IVA = ivacaftor; LUM = lumacaftor; TEZ = tezacaftor; ^2 = twice a day.

ppFEV_1_: Of all the CFTR modulators examined to date, individuals with a G551D mutation treated with IVA experienced the largest improvement in ppFEV_1_ compared to placebo (n = 2 studies; n = 213; weighted absolute mean difference 10.8, 95% CI: 9.0–12.7) (Fig. 3A) with no heterogeneity (I^2^ = 0%) in results between studies (Fig. 3B)^20,21^.

For F508del homozygous individuals 12 years and older, ppFEV_1_ significantly improved with LUM-IVA and TEZ-IVA compared to placebo (Fig. 3A). The effect size was similar for TEZ-IVA (n = 2 studies; n = 535; weighted absolute mean difference 4.0, 95% CI: 3.2–4.8)^22,23^ and higher dose LUM-IVA (n = 3 studies; n = 755; weighted absolute mean difference 3.4, 95% CI: 2.4–4.4) (Fig. 3B)^17,24^. For individuals 6–11 years, there was a mild increase in ppFEV_1_ for LUM-IVA compared to placebo (n = 1 study; n = 204; absolute mean difference 2.4, 95% CI: 0.4–4.4)^19^. No significant treatment effect was observed with IVA or TEZ alone, and there was a trend toward worsening in ppFEV_1_ for F508del homozygous individuals treated with higher doses of LUM (Fig. 3A)^22,24,25^.

For F508del heterozygous individuals, there was no significant improvement in ppFEV_1_ on LUM or LUM-IVA (Fig. 3A)^24,26^. In a small study involving individuals with F508del/G551D, TEZ-IVA did not lead to a significant improvement in ppFEV_1_ compared to IVA alone^22^.

For individuals with the R117H mutation on at least one allele, IVA did not lead to an overall improvement in ppFEV_1_ compared to placebo, but there was a significant improvement in a pre-defined subgroup analysis restricted to adults (n = 50; absolute mean difference 5.0, 95% CI 1.2–8.8)^27^. For individuals with a nonsense mutation on at least one allele, ataluren did not result in a significant relative improvement in ppFEV_1_ compared to placebo^18^.

#### Secondary outcomes

Pulmonary exacerbations (PEx): Eight studies examined *protocol-defined* PEx as described in Appendix Table 3. Of all the CFTR modulators examined, individuals (≥12 years old) with a G551D mutation receiving IVA derived the greatest reduction in PEx risk compared to placebo (n = 1 study; n = 161; OR 0.39, 95% CI: 0.21–0.74) (Appendix Fig. 2A)^20^. LUM-IVA and TEZ-IVA also significantly reduced the risk of PEx compared to placebo in F508del homozygous individuals (≥12 years old) but the risk reduction was less than that observed with IVA in G551D (Appendix Fig. 2A,B)^17,23^. In comparison to placebo, no significant reduction in PEx risk was observed for F508del homozygous individuals or individuals with the R117H mutation on at least one allele receiving IVA, nor for individuals with a nonsense mutation receiving ataluren (Appendix Fig. 2A)^18,25,27^.

Pulmonary exacerbations (PEx) requiring hospitalization: LUM-IVA reduced the risk of PEx requiring hospitalization in F508del homozygous individuals (Appendix Fig. 3A,B)^17^. TEZ-IVA also significantly reduced the rate of PEx leading to hospitalization compared to placebo (n = 1 study; n = 504; rate ratio 0.53, 95% CI 0.34–0.82) but a risk ratio could not be calculated^23^. Individuals with the G551D mutation on at least one allele treated with IVA also experienced a reduction in the risk of PEx requiring hospitalization but this was not statistically significant (Appendix Fig. 3A)^20^.

CFQ-R respiratory domain: Compared to placebo, CFQ-R Respiratory domain scores improved to a similar extent for IVA treated individuals (≥6 years old) with the G551D mutation on at least one allele (n = 3 studies; n = 236; weighted absolute mean difference: 7.2, 95% CI: 3.3–11.1)^20,21,28^, IVA treated individuals ≥18 years old with at least one R117H mutation (n = 1 study; n = 69; absolute mean difference: 8.4, 95% CI: 2.2–14.6)^27^, and for LUM-IVA treated F508del heterozygous individuals ≥18 years old (n = 1 study; n = 125; absolute mean difference: 6.5, 95% CI 1.4–11.6) (Appendix Fig. 4A,B). CFQ-R Respiratory domain scores also significantly improved with TEZ-IVA and LUM-IVA in F508del homozygous individuals (≥12 years old) but the mean difference did not exceed the minimal clinically important difference (MCID) for LUM-IVA^17,23,24^. Furthermore, there was no significant improvement in CFQ-R Respiratory domain scores for patients 6–11 years old on LUM-IVA compared to placebo^19^.

There was worsening of the CFQ-R Respiratory domain score for F508del homozygous and heterozygous individuals (≥18 years old) on LUM alone (Appendix Fig. 4A)^24^. In a small phase 2 study involving individuals with F508del/G551D, TEZ-IVA did not lead to significant improvement in the CFQ-R Respiratory domain compared to IVA alone^22^. For individuals with a nonsense mutation on at least one allele, ataluren did not modify CFQ-R Respiratory domain score compared to placebo^18^.

Nutritional outcomes (BMI and weight): For individuals with at least one G551D mutation (≥6 years old), significant improvements in weight were observed on IVA compared to placebo (n = 2 studies; n = 213; weighted absolute mean difference: 2.8 kg, 95% CI: 1.8–3.8) (Appendix Fig. 5A,B)^20,21^. For F508del homozygous individuals (≥12 years old), a clinically modest but statistically significant increase in BMI was observed for both doses of LUM-IVA compared to placebo (Appendix Fig. 6A,B)^17^; however, no significant treatment effect was seen in individuals 6–11 years on LUM-IVA (Appendix Fig. 6A)^19^. TEZ-IVA did not lead to improvement in BMI compared to placebo in individuals 12 years and older (Appendix Fig. 6A)^23^. For F508del heterozygous individuals (≥18 years old), LUM-IVA did not result in significant improvement in weight or BMI compared to placebo^26^. There were no significant improvements in BMI compared to placebo among IVA treated individuals with an R117H mutation (Appendix Fig. 6A**)** or ataluren treated individuals with a nonsense mutation (data not shown)^18,27^.

Adverse event reporting: CFTR modulators were generally well tolerated compared to placebo (Appendix Figs 7–30**)**. For studies involving F508del homozygous and heterozygous individuals, those assigned to LUM had increased dyspnea and “abnormal respiration” compared to placebo (Appendix Figs 11 and 13). F508del homozygous and heterozygous subjects assigned to LUM and LUM-IVA also had more respiratory-related adverse events leading treatment discontinuation compared to placebo (Appendix Table 4)^17,24^. For the one study involving individuals with a nonsense mutation, subjects receiving ataluren had increased incidence of acute kidney injury compared to placebo (15% vs. <1%) resulting in higher rates of treatment discontinuation^18^.

The prevalence of LFT abnormalities was generally similar between treatment and placebo, however there were a few exceptions. A greater proportion of G551D patients had severe ALT elevations (>8x ULN) on IVA compared to placebo (3.6% vs 0%) (Appendix Table 5)^20^. Milder elevations in AST (2–3X ULN) were observed for G551D patients on IVA and ALT or AST (>3X ULN) in F508del homozygous children aged 6–11 on LUM-IVA compared to placebo (Appendix Table 5)^19,20^.

Level of agreement for study selection and data extraction: There was a strong level of agreement (95%) for the articles selected between the two reviewers for full text review and 100% agreement between the two reviewers for the articles meeting eligibility criteria for inclusion in this review. The level of agreement for data extraction were as follows: study characteristics (n = 88 data points, 95% agreement), risk of bias (n = 92 data points, 84% agreement), and effects of the intervention (n = 480 data points, 81% agreement).

## Discussion

This study represents the most comprehensive systematic review of the efficacy and safety of CFTR modulators performed to date. While evidence-based recommendations for the use of CFTR modulators were recently published and provides a valuable resource for practicing clinicians, this review provides a more concise and up-to-date synthesis of all the placebo-controlled clinical trial data^29^. No prior systematic review has compared all investigational CFTR modulators from phase 2 and 3 RCTs in specific CF genotypes^30–32^.

As this review highlights, patients with gating mutations such as G551D benefit the most from current CFTR modulators and those that are F508 homozygous have moderate benefit in comparison. Based on published parallel design trials, CFTR modulators have not been effective in F508 heterozygotes or those with nonsense mutations. However, in a recent phase 3 cross-over study evaluating IVA and TEZ-IVA in individuals ≥12 years old with F508del and a residual CFTR function mutation, improvements in ppFEV1 of 4.7% and 6.8%, respectively, were observed compared to placebo^33^. Furthermore, unpublished phase 2 data evaluating TEZ-IVA in combination with “next-generation” corrector molecules have demonstrated significant improvements in ppFEV1 in subjects with F508del and a minimal CFTR function mutation, some of whom have nonsense mutations.

When comparing the efficacy of CFTR modulators across all genotypes for ppFEV_1_, CF individuals (≥6 years old) with the G551D mutation on at least one allele receiving IVA experienced the largest benefit^20,21^. F508del homozygous subjects receiving TEZ-IVA (≥12 years old) and LUM-IVA (≥6 years old) also had improvements in ppFEV_1_ compared to placebo but the effect sizes were modest compared to IVA in G551D^17,19,24^. Individuals (≥18 years old) with the R117H mutation on at least one allele treated with IVA experienced similar improvement in ppFEV_1_ to F508del homozygous subjects treated with TEZ-IVA and LUM-IVA.

Similar to ppFEV_1_, the effect of CFTR modulators on PEx risk and respiratory symptoms were most pronounced with IVA in G551D adolescents and adults (≥12 years old), with a 60% reduction in PEx risk and a 7-point improvement in the CFQ-R Resp domain^20,21^. F508del homozygous adolescents and adults also had a 40–45% reduction in PEx risk on TEZ-IVA and LUM-IVA. While F508del homozygous subjects experienced improvements in the CFQ-R Resp domain on both TEZ-IVA and LUM-IVA, this was not clinically significant for LUM-IVA. Individuals with a R117H mutation also experienced improvements in the CFQ-R Resp domain on IVA, with a magnitude of change in the adults comparable to that observed with IVA in G551D. The effect of CFTR modulators on weight were most significant with IVA in G551D individuals (≥6 years old). While F508del homozygous individuals (≥12 years old) had improvement in BMI with LUM-IVA, the effect size was modest.

Most of the CFTR modulator therapies examined in this review were well tolerated with the exception of increased reporting of respiratory adverse events (e.g. dyspnea) leading to higher rates of treatment discontinuation in patients randomized to LUM and LUM-IVA. The molecular mechanism responsible for the adverse respiratory effects (e.g. dyspnea, abnormal respiration) for patients on LUM remain unclear but appears to be an off-target effect specific to LUM, as opposed to being related to F508del CFTR correction per se, as similar adverse effects have not been observed with F508del CFTR correction with TEZ-IVA^22,23,34^. There was also increased reporting of acute kidney injury for nonsense mutation patients assigned to ataluren compared to placebo. The long-term safety of CFTR modulator therapies beyond one year could not be assessed in this review and therefore the detection of infrequent or long-term side effects will require ongoing post-marketing surveillance^35,36^.

There are several potential limitations of this review. We excluded cross-over, open-label, and observational studies to avoid carryover effects and to ensure we incorporated the highest level of evidence. We also limited our inclusion to full-text studies which could have resulted in publication bias. We focused on pre-defined clinically important outcomes but did not include multiple-breath washout measurement (e.g. LCI_2.5_) given the lack of clinical trials utilizing this outcome measure^19^.

There remain several gaps in the placebo-controlled evidence base for CFTR modulators. RCTs to date have excluded young children (<6 years old) and therefore the earliest age of safe use of CFTR modulators remains uncertain. However, small open-label 24-week studies have demonstrated a similar safety profile of IVA in children 1–5 years old with CFTR gating mutations compared to older age groups studied^37,38^. Most RCTs have also excluded CF individuals with severe lung disease (ppFEV_1_ < 40%), individuals colonized/infected with bacteria associated with rapid lung function decline (e.g. *Burkholderia cenocepacia*, *Mycobacterium abscessus*), and individuals with very frequent pulmonary exacerbations requiring continuous or near continuous systemic antibiotics by virtue of requiring clinical stability and no systemic antibiotics 4 weeks prior to randomization and therefore the efficacy and safety of CFTR modulators in these sub-groups remain unclear. For example, based on observational data, F508del homozygous individuals with advanced lung disease started on LUM-IVA have increased respiratory-related adverse events leading to treatment discontinuation; therefore, closer monitoring following treatment initiation is recommended^39,40^.

Most placebo-controlled RCTs to date have been limited to a maximum duration of 48 weeks and therefore the long-term placebo-controlled effects of these therapies remain unclear. However, an open-label extension trial evaluating the long-term effects of ivacaftor up to 144 weeks has demonstrated sustained clinical benefits of ivacaftor on lung function, weight, patient-reported respiratory symptoms and PEx risk reduction with no new safety concerns^35^. Furthermore, based on combined data from an open-label extension trial and U.S. CF patient registry data, the rate of lung function decline over 3 years was lower in G551D patients treated with ivacaftor compared to propensity-matched controls from the CF registry, suggestive of a disease-modifying effect over the longer term.

In conclusion, based on randomized placebo-controlled parallel design trials, CFTR potentiation with IVA in individuals with a G551D mutation is safe, and results in robust clinical benefits compared to placebo and to date is superior to the effects observed with CFTR modulators in other CF genotypes. The effects of TEZ-IVA and LUM-IVA in F508del homozygous individuals are comparable with respect to the magnitude of change in ppFEV_1_ and PEx risk reduction but TEZ-IVA is safer and leads to greater improvement in respiratory symptoms.

## Supplementary information

Online Appendix
