# Supplementary material for: A Systematic Review of the Clinical Efficacy and Safety of CFTR Modulators in Cystic Fibrosis
Source: Sci Rep. 2019 May 10;9:7234. doi: 10.1038/s41598-019-43652-2 (PMC6510767; doi:10.1038/s41598-019-43652-2)
Supplement: Supplementary file 1 — Online Appendix [file 41598_2019_43652_MOESM1_ESM.docx]

**Online Appendix: A Systematic Review of the Clinical Efficacy and Safety of CFTR Modulators in Cystic Fibrosis**

**Authors:**

Al-Rahim R. Habib^1^

Majid Kajbafzadeh^1^

Sameer Desai^2^

Connie Yang^3^

Kate Skolnik^4^

Bradley S. Quon^5^

**Affiliations:**

^1^ School of Medicine, The University of Sydney

^2^ School of Population and Public Health, University of British Columbia

^3^ Division of Respiratory Medicine, Department of Pediatrics, University of British Columbia

^4^ Division of Respirology, Department of Medicine, University of Calgary

^5^ Centre for Heart Lung Innovation, St. Paul’s Hospital, Department of Medicine, University of British Columbia

**Corresponding Author:**

Bradley S. Quon

#166 – 1081 Burrard St.

Vancouver, BC

V6Z 1Y6

Canada

**Shortened Title:** Systematic review of CFTR Modulators

**Authors’ contributions:** AH, MK, BSQ contributed to all aspects of this study including study concept and design, conducting the literature search, study design, data collection, data analysis, data interpretation, and writing of the manuscript. SD, CY, KS contributed to data analysis, data interpretation, and writing of the manuscript.

**Conflict of Interest Statement:** BSQ has received consulting fees from Proteostasis Therapeutics Inc. and Horizon Pharma and has served as site PI for Vertex sponsored clinical trials.

**Funding:** No grants or third-party funding was provided for this study.

**Ethics Committee Approval:** N/A

*Statistical analysis*

Continuous outcomes were analyzed as absolute change in values pre- *versus* post-treatment between the experimental and control groups. Dichotomous outcomes were analyzed using odds ratios (OR).

Meta-analysis utilized fixed effects for the statistical model using Review Manager (Version 5.3; The Cochrane Collaboration, 2014). Heterogeneity in the results across trials was assessed using the I^2^-statistic. The I^2^ statistic describes the percentage of variability in effect estimates due to heterogeneity rather than sampling error (chance) and is classified according to the Cochrane Handbook: 0% to 40%: might not be important; 30% to 60%: may represent moderate heterogeneity; 50% to 90%: may represent substantial heterogeneity; and 75% to 100%: considerable heterogeneity.^1^

Absolute changes were derived from the mean pre- and post-treatment values, if not reported in the study text or supplementary data. Standard errors (SE) were calculated from reported confidence intervals or p-values using calculations provided in the Cochrane Handbook of Systematic Reviews of Interventions.^1^ If confidence intervals or p-values were not reported, effect estimates could not be derived.

**Appendix Table-1. Age, ppFEV1, and Sub-groups of Included Studies.**

| **Genotype** | **First Author and Year** | **Phase** | **Age** | **Age subgroups** | **ppFEV_1_** | **ppFEV_1_ subgroups** |
| --- | --- | --- | --- | --- | --- | --- |
| F508del homozygous | Flume (2012)^2^ | 2 | ≥12 | NR | ≥40 | NR |
|  | Clancy (2012)^3^ | 2 | ≥18 | NR | ≥40 | NR |
|  | Boyle (2014)^4^ | 2 | ≥18 | NR | ≥40 | NR |
|  | Donaldson (2018)^5^ | 2 | ≥18 | NR | 40-90 | NR |
|  | Wainwright (2015)^6^ | 3 | ≥12 | ≥12 to <18 vs. ≥18 | 40-90 | <40 vs. ≥40  <70 vs. ≥70 |
|  | Ratjen (2017)^7^ | 3 | 6-11 | NR | ≥70 | NR |
|  | Taylor-Cousar (2017)^8^ | 3 | ≥12 | ≥12 to <18 vs. ≥18 | 40-90 | <40 vs. ≥40 to <70 vs. ≥70 |
| F508del heterozygous | Boyle (2014)^4^ | 2 | ≥18 | NR | ≥40 | NR |
|  | Rowe (2016)^9^ | 2 | ≥18 | NR | 40-90 | NR |
| F508del/G551D | Donaldson (2018)^5^ |  | ≥12 | NR |  | NR |
| G511D | Accurso (2010)^10^ | 2 | ≥18 | NR | ≥40 | NR |
|  | Ramsey (2011)^11^ | 3 | ≥12 | <18 vs. ≥18 | 40-90 | <70 vs. ≥70* |
|  | Davies (2013)^12^ | 3 | 6-11 | NR | 40-105 | <=90 vs. >90* |
| R117H | Moss (2015)^13^ | 3 | 6-11  ≥12 | 6-11 vs. 12-17 vs. ≥18 | 40-105  40-90 | <70 vs. ≥70 to <=90 vs. >90 |
| Nonsense mutation | Kerem (2014)^14^ | 3 | ≥6 | <18 vs. ≥18 | 40-90 | 40 to <65 vs. ≥65 to 90 |

**Abbreviations:** ppFEV_1_= percent-predicted forced expiratory volume in 1 second; NR=not reported

*Post-hoc analysis combining Ramsey and Davies was not included (evaluated subgroup with ppFEV_1_ 50-80)

**Appendix Table-2. *A Priori* Clinical Outcomes of Interest.**

| **Outcomes of Interest** | **Number of Studies** | |
| --- | --- | --- |
|  | **Phase 2** | **Phase 3** |
| ppFEV_1_* | 5 | 8 |
| Pulmonary exacerbations^ | 1 | 8 |
| Hospitalization for pulmonary exacerbations^∞^ | 1 | 3 |
| CFQ-R Respiratory domain^#^ | 6 | 8 |
| BMI^Ψ^ | 1 | 6 |
| Weight^δ^ | 2 | 7 |

**Footnotes:**

*11 of 13 studies were included in Figure-3A. Two studies were excluded (Accurso 2010^10^, Kerem 2014^14^) as they examined relative and not absolute change in ppFEV_1_.

^6 of 9 studies were included in Appendix Figure-2A. One study was excluded as there was no exacerbation reporting (Ratjen 2017^7^), one study had a low number of events (Davies 2013^12^), and one study reported mean exacerbation events (Kerem 2014^14^).

^∞^3 of 4 studies were included in Appendix Figure-3A as one study did not report hospitalizations although this was described in the methods as an endpoint (Flume 2012^2^).

^#^12 of 14 studies were included in Appendix Figure-4A. Two studies were excluded as only the mean effect was reported without variance measure or p-value (Flume 2012^2^, Clancy 2012^3^).

^Ψ^6 of 7 studies were included in Appendix Figure-5A. One study was excluded as only mean effect was reported and the result was reported as not statistically significant but without variance measure or p-value (Kerem 2014^14^).

^δ^3 of 9 studies could be included in Appendix Figure-6A. BMI but not weight was reported in 4 studies (Wainwright 2015 x 2^6^, Ratjen 2017^7^, Taylor-Cousar^8^) and only the mean effect was reported without variance measures or p-value for 2 studies (Flume 2012^2^, Kerem 2014^14^).

**Abbreviations:** BMI=body mass index; CFQ-R=Cystic Fibrosis Questionnaire-Revised; ^7,9^ppFEV_1_= percent-predicted forced expiratory volume in 1 second

### **Appendix Table-3. Definition of Protocol-defined Pulmonary Exacerbations for Included Studies.**

| **First Author and Year** | **Phase** | **Outcome** | **Study Duration** | **Protocol-defined exacerbation criteria** |
| --- | --- | --- | --- | --- |
| Ramsey (2011)^11^ | 3 | Secondary, Tertiary | 48 | Modified Fuchs |
| Flume (2012)^2^ | 2 | Tertiary | 16 | Modified Fuchs |
| Davies (2013)^12^ | 3 | Secondary^†^ | 48 | Modified Fuchs |
| Kerem (2014)^14^ | 3 | Secondary | 48 | Modified Fuchs |
| Moss (2015)^13^ | 3 | Secondary, Tertiary | 24 | Modified Fuchs |
| Wainwright (2015)^6^ | 3 | Secondary | 24 | Modified Fuchs |
| Ratjen (2017)^7^ | 3 | Secondary | 24 | Modified Fuchs |
| Taylor-Cousar (2017)^8^ | 3 | Secondary | 24 | Modified Fuchs |

^†^protocol-defined exacerbation rate was low (4 events for ivacaftor and 3 events for placebo) and therefore was not analyzed statistically.

**Appendix Table-4. Treatment Discontinuations due to Adverse Events.**

| **Genotype** | **First Author** | **Drug** | **Dose (mg)** | **Experimental** | | | | **Placebo** | | | |
| --- | --- | --- | --- | --- | --- | --- | --- | --- | --- | --- | --- |
|  |  |  |  | **Total** | **Events** | **%** | **AE leading to discontinuation** | **Total** | **Events** | **%** | **AE leading to discontinuation** |
| F508del homozygous | Flume (2012)^2^ | IVA | 150^2 | 112 | 3 | 2.7 | Fatigue (1)  Arthritis (1)  Myopathy (1) | 28 | 2 | 7.1 | Cognitive disorder (1)  ALT/AST > 8X ULN (1) |
|  | Clancy (2012)^3^ | LUM | 25 | 18 | 1 | 5.6 | Respiratory AE (1) | 17 | 0 | 0 | - |
|  |  |  | 50 | 18 | 1 | 5.6 | Respiratory AE (1) | 17 | 0 | 0 | - |
|  |  |  | 100 | 17 | 1 | 5.9 | Respiratory AE (1) | 17 | 0 | 0 | - |
|  |  |  | 200 | 19 | 1 | 5.3 | Respiratory AE (1) | 17 | 0 | 0 | - |
|  | Boyle (2014)^4^ | LUM | 200 (Cohort 1a + 1b) | 41 | 1 | 2.4 | Chest tightness (1) | 21 | 0 | 0 | - |
|  |  |  | 200 (Cohort 2) | 23 | 2 | 8.7 | “Adverse event” (2) | 23 | 1 | 4.3 | Required prohibited med (1) |
|  |  |  | 400 (Cohort 2) | 21 | 1 | 4.8 | “Adverse event” (1) |  |  |  |  |
|  |  |  | 600 (Cohort 2) | 21 | 1 | 4.8 | “Adverse event” (1) |  |  |  |  |
|  |  |  | 400^2 (Cohort 3) | 11 | 1 | 9.1 | “Adverse event” (1) | 4 | 1 | 25.0 | Required prohibited med (1) |
|  | Boyle (2014)^4^ | LUM-IVA | 200 + 150^2 (Cohort 1a) | 20 | 0 | 0 | - | 21 | 0 | 0 | - |
|  |  |  | 200 + 250^2 (Cohort 1b) | 21 | 0 | 0 | - |  |  |  |  |
|  |  |  | 200 + 250^2 (Cohort 2) | 21 | 0 | 0 | - | 23 | 0 | 0 | - |
|  |  |  | 400 + 250^2 (Cohort 2) | 20 | 0 | 0 | - |  |  |  |  |
|  |  |  | 600 + 250^2 (Cohort 2) | 20 | 0 | 0 | - |  |  |  |  |
|  |  |  | 400^2 + 250^2 (Cohort 3) | 11 | 0 | 0 | - | 4 | 0 | 0 | - |
|  | Wainwright (2015)^6^ | LUM-IVA | 600 + 250^2 | 369 | 14 | 3.8 | Adverse events observed in ≥2 patients (both doses combined):  Elevated CK (4)  Hemoptysis (3)  Bronchospasm (2)  Dyspnea (2)  Exacerbation (2)  Rash (2) | 370 | 6 | 1.6 | No adverse events were observed in ≥ 2 patients |
|  |  |  | 400^2 + 250^2 | 369 | 17 | 4.6 |  |  |  |  |  |
|  | Ratjen (2017)^7^ | LUM-IVA | 100^2 + 125^2 | 103 | 6 | 5.8 | Adverse events (2)  Respiration abnormal (1) | 101 | 5 | 5.0 | Adverse events (2) |
|  | Donaldson (2018)^5^ | TEZ | 10 | 8 | 1 | 12.5 | “Adverse event” (1) | 33 | 0 | 0 | - |
|  |  |  | 30 | 8 | 0 | 0 | - |  |  |  |  |
|  |  |  | 100 | 8 | 0 | 0 | - |  |  |  |  |
|  |  |  | 150 | 9 | 0 | 0 | - |  |  |  |  |
|  | Donaldson (2018)^5^ | TEZ-IVA  (dose escalation) | 10 + 150^2 | 18 | 0 | 0 | - |  |  |  |  |
|  |  |  | 30 + 150^2 | 19 | 0 | 0 | - |  |  |  |  |
|  |  |  | 100 + 150^2 | 17 | 0 | 0 | - |  |  |  |  |
|  |  |  | 150 + 150^2 | 17 | 0 | 0 | - |  |  |  |  |
|  | Donaldson (2018)^5^ | TEZ-IVA  (dose testing) | 50^2 + 150^2 | 16 | 0 | 0 | - |  |  |  |  |
|  |  |  | 100 + 50^2 | 19 | 0 | 0 | - |  |  |  |  |
|  |  |  | 100 + 150^2 | 17 | 0 | 0 | - |  |  |  |  |
|  | Taylor-Cousar (2017)^8^ | TEZ-IVA | 100 + 150^2 | 251 | 7 | 2.8 | Adverse events observed in ≥2 patients:  Abdominal pain (2)  Increased AST/ALT (2) | 258 | 8 | 3.1 | Adverse events observed in ≥2 patients:  Increased AST/ALT (2)  Increased ALP (2)  Fatigue (2)  Headache (2) |
| F508del heterozygous | Boyle (2014)^4^ | LUM | 600 | 21 | 2 | 9.5 | “Adverse event” (2) | 23 | 1 | 4.3 | Required prohibited med (1) |
|  | Boyle (2014)^4^ | LUM-IVA | 600 + 250^2 | 21 | 2 | 9.5 | Required prohibited med (1)  Withdrew consent (1) | 23 | 0 | 0 | - |
|  | Rowe (2017)^9^ | LUM-IVA | 400^2 + 250^2 | 62 | 4 | 6.5 | Exacerbation (2)  Increased AST/ALT (1)  Abnormal respiration (1) | 63 | 0 | 0 | - |
| F508del/G551D | Donaldson (2018)^5^ | TEZ-IVA | 100 + 150^2 | 14 | 0 | 0 | - | 4* | 0 | 0 | - |
| G551D | Accurso (2010)^10^ | IVA | 150^2 (Part 2) | 8 | 0 | 0 | - | 4 | 0 | 0 | - |
|  |  |  | 250^2 (Part 2) | 7 | 0 | 0 | - | 4 | 0 | 0 | - |
|  | Davies (2013)^12^ | IVA | 150^2 | 26 | 0 | 0 | - | 26 | 1 | 3.8 | Anxiety (1) |
|  | Ramsey (2011)^11^ | IVA | 150^2 | 83 | 1 | 1.2 | Increased LFTs | 78 | 4 | 5.1 | Increased LFTs (1)  AV block (1)  Respiratory failure (1)  Panic attack (1) |
| R117H | Moss (2015)^13^ | IVA | 150^2 | 34 | 0 | 0 | - | 35 | 0 | 0 | - |
| Nonsense mutation | Kerem (2014)^14^ | Ataluren | 10/10/20 mg/kg | 120 | 8 | 6.7 | AKI (4)  Rash (1)  CFRD (1)  Pancreatitis (1)  Diarrhea (1)  UTI (1)  Pyrexia (1)  Exacerbation (1)  Nephrolithiasis (1) | 118 | 3 | 2.5 | Abdominal pain (2)  Headache (1)  Hemoptysis (1) |

*****Placebo group included ivacaftor 150^2

**Abbreviations:** AE=adverse event; AKI=acute kidney injury; ALT=alanine aminotransferase; AST=aspartate aminotransferase; AV=atrioventricular; CFRD=CF-related diabetes; CK=creatine kinase; IVA=ivacaftor; LFT=liver function test; LUM=lumacaftor; MVA=motor vehicle accident; TEZ=tezacaftor; ULN=upper limit of normal; UTI=urinary tract infection.

**Appendix Table-5. Frequency of Liver Function Test Abnormalities.**

| **Genotype** | **First Author** | **Drug** | **Dose (mg)** | **Study Duration (weeks)** | **LFT abnormality** | **Experimental** | | | **Control** | | |
| --- | --- | --- | --- | --- | --- | --- | --- | --- | --- | --- | --- |
|  |  |  |  |  |  | **Total** | **Events** | **%** | **Total** | **Events** | **%** |
| F508del homozygous | Flume (2012)^2^ | IVA | 150^2 | 16 | ALT or AST > 3x ULN | 112 | 4 | 3.6 | 28 | 2 | 7.1 |
|  |  |  |  |  | ALT or AST > 8x ULN | 112 | 0 | 0 | 28 | 1 | 3.6 |
|  | Clancy (2012)^3^ | LUM | 25 | 4 | “No clinically significant changes in lab findings during the study” | 18 | 0 | 0 | 17 | 0 | 0 |
|  |  |  | 50 | 4 |  | 18 | 0 | 0 | 17 | 0 | 0 |
|  |  |  | 100 | 4 |  | 17 | 0 | 0 | 17 | 0 | 0 |
|  |  |  | 200 | 4 |  | 19 | 0 | 0 | 17 | 0 | 0 |
|  | Boyle (2014)^4^ | LUMA | 200 (Cohort 1a + 1b) | 2 | No LFT abnormalities reported | 41 | 0 | 0 | 21 | 0 | 0 |
|  |  |  | 200 (Cohort 2) | 4 |  | 23 | 0 | 0 | 23 | 0 | 0 |
|  |  |  | 400 (Cohort 2) | 4 |  | 21 | 0 | 0 |  |  |  |
|  |  |  | 600 (Cohort 2) | 4 |  | 21 | 0 | 0 |  |  |  |
|  |  |  | 400^2 (Cohort 3) | 4 |  | 11 | 0 | 0 | 4 | 0 | 0 |
|  | Boyle (2014)^4^ | LUM-IVA | 200 + 150^2 (Cohort 1a) | 1 | No LFT abnormalities reported | 20 | 0 | 0 | 21 | 0 | 0 |
|  |  |  | 200 + 250^2 (Cohort 1b) | 1 |  | 21 | 0 | 0 |  |  |  |
|  |  |  | 200 + 250^2 (Cohort 2) | 4 |  | 21 | 0 | 0 | 23 | 0 | 0 |
|  |  |  | 400 + 250^2 (Cohort 2) | 4 |  | 20 | 0 | 0 |  |  |  |
|  |  |  | 600 + 250^2 (Cohort 2) | 4 |  | 20 | 0 | 0 |  |  |  |
|  |  |  | 400^2 + 250^2 (Cohort 3) | 4 |  | 11 | 0 | 0 | 4 | 0 | 0 |
|  | Wainwright (2015)^6^ | LUM-IVA | 600 + 250^2 | 24 | ALT >3x to <=5x ULN | 369 | 13 | 3.5 | 370 | 15 | 4.1 |
|  |  |  |  |  | AST >3x to <=5x ULN | 369 | 8 | 2.2 | 370 | 4 | 1.1 |
|  |  |  |  |  | ALT >5x to <=8x ULN | 369 | 4 | 1.1 | 370 | 1 | 0.3 |
|  |  |  |  |  | AST >5x to <=8x ULN | 369 | 4 | 1.1 | 370 | 5 | 1.4 |
|  |  |  |  |  | ALT >8x ULN | 369 | 3 | 0.8 | 370 | 0 | 0 |
|  |  |  |  |  | AST >8x ULN | 369 | 3 | 0.8 | 370 | 2 | 0.5 |
|  |  |  |  |  | Total bilirubin >1.5x ULN | 369 | 0 | 0 | 370 | 5 | 1.4 |
|  |  |  |  |  | Total bilirubin >2x ULN | 369 | 2 | 0.5 | 370 | 1 | 0.3 |
|  |  |  | 400^2 + 250^2 | 24 | ALT >3x to <=5x ULN | 369 | 8 | 2.2 | 370 | 15 | 4.1 |
|  |  |  |  |  | AST >3x to <=5x ULN | 369 | 7 | 1.9 | 370 | 4 | 1.1 |
|  |  |  |  |  | ALT >5x to <=8x ULN | 369 | 1 | 0.3 | 370 | 1 | 0.3 |
|  |  |  |  |  | AST >5x to <=8x ULN | 369 | 2 | 0.5 | 370 | 5 | 1.4 |
|  |  |  |  |  | ALT >8x ULN | 369 | 1 | 0.3 | 370 | 0 | 0 |
|  |  |  |  |  | AST >8x ULN | 369 | 2 | 0.5 | 370 | 2 | 0.5 |
|  |  |  |  |  | Total bilirubin >1.5x ULN | 369 | 0 | 0 | 370 | 5 | 1.4 |
|  |  |  |  |  | Total bilirubin >2x ULN | 369 | 1 | 0.3 | 370 | 1 | 0.3 |
|  | Ratjen (2017)^7^ | LUM-IVA | 100^2 + 125^2 | 24 | ALT or AST > 3x ULN | 103 | 13 | 12.6 | 101 | 8 | 7.9 |
|  |  |  |  |  | ALT or AST > 5x ULN | 103 | 5 | 4.9 | 101 | 3 | 3.0 |
|  |  |  |  |  | ALT or AST > 8x ULN | 103 | 1 | 1.0 | 101 | 2 | 2.0 |
|  | Donaldson (2018)^5^ | TEZ | 10 | 4 | "No clinically significant trends for laboratory tests” | 8 | NR | NR | 33 | NR | NR |
|  |  |  | 30 |  |  | 8 | NR | NR |  | NR | NR |
|  |  |  | 100 |  |  | 8 | NR | NR |  | NR | NR |
|  |  |  | 150 |  |  | 9 | NR | NR |  | NR | NR |
|  |  | TEZ-IVA | 10 + 150^2 | 4 |  | 18 | NR | NR |  | NR | NR |
|  |  |  | 30 + 150^2 |  |  | 19 | NR | NR |  | NR | NR |
|  |  |  | 100 + 150^2 |  |  | 17 | NR | NR |  | NR | NR |
|  |  |  | 150 + 150^2 |  |  | 17 | NR | NR |  | NR | NR |
|  |  | TEZ-IVA | 50^2 + 150^2 | 4 |  | 16 | NR | NR |  | NR | NR |
|  |  |  | 100 + 50^2 |  |  | 19 | NR | NR |  | NR | NR |
|  |  |  | 100 + 150^2 |  |  | 17 | NR | NR |  | NR | NR |
|  | Taylor-Cousar (2017)^8^ | TEZ-IVA | 100 + 150^2 | 24 | ALT >3x to <=5x ULN | 249 | 7 | 2.8 | 257 | 9 | 3.5 |
|  |  |  |  |  | AST >3x to <=5x ULN | 249 | 7 | 2.8 | 257 | 3 | 1.2 |
|  |  |  |  |  | ALT >5x to <=8x ULN | 249 | 1 | 0.4 | 257 | 3 | 1.2 |
|  |  |  |  |  | AST >5x to <=8x ULN | 249 | 0 | 0 | 257 | 2 | 0.8 |
|  |  |  |  |  | ALT >8x ULN | 249 | 0 | 0 | 257 | 0 | 0 |
|  |  |  |  |  | AST >8x ULN | 249 | 0 | 0 | 257 | 2 | 0.8 |
|  |  |  |  |  | Total bilirubin >1.5x to <=2x ULN | 249 | 5 | 2.0 | 257 | 5 | 1.9 |
|  |  |  |  |  | Total bilirubin >2x to <=3x ULN | 249 | 2 | 0.8 | 257 | 5 | 1.9 |
|  |  |  |  |  | Total bilirubin >3x ULN | 249 | 2 | 0.8 | 257 | 1 | 0.4 |
| F508del heterozygous | Boyle (2014)^4^ | LUM | 600 | 4 | No post-treatment LFT abnormalities reported | 21 | 0 | 0 | 23 | 0 | 0 |
|  | Boyle (2014)^4^ | LUM-IVA | 600 + 250^2 | 4 | No LFT abnormalities reported | 21 | 0 | 0 | 23 | 0 | 0 |
|  | Rowe (2017)^9^ | LUM-IVA | 400^2 + 250^2 | 8 | 1 patient assigned to LUM-IVA discontinued treatment due to increased AST/ALT | 62 | NR | NR | 63 | NR | NR |
| F508del/G551D | Donaldson (2018)^5^ | TEZ-IVA | 100 + 150^2 | 4 | "No clinically significant trends for laboratory tests” | 14 | NR | NR | 4* | NR | NR |
| G551D | Accurso (2011)^10^ | IVA | 150^2 (Part 2) | 4 | No LFT abnormalities reported | 8 | 0 | 0 | 4 | 0 | 0 |
|  |  |  | 250^2 (Part 2) | 4 | No LFT abnormalities reported | 7 | 0 | 0 | 4 | 0 | 0 |
|  | Davies (2013)^12^ | IVA | 150^2 | 48 | “ALT increased” | 26 | 2 | 7.7 | 26 | 3 | 11.5 |
|  | Ramsey (2011)^11^ | IVA | 150^2 | 48 | ALT >2x to <=3x ULN | 83 | 5 | 6.0 | 78 | 6 | 7.7 |
|  |  |  |  |  | AST >2x to <=3x ULN | 83 | 8 | 9.6 | 78 | 4 | 5.1 |
|  |  |  |  |  | ALT >3x to <=5x ULN | 83 | 0 | 0 | 78 | 2 | 2.6 |
|  |  |  |  |  | AST >3x to <=5x ULN | 83 | 1 | 1.2 | 78 | 2 | 2.6 |
|  |  |  |  |  | ALT >5x to <=8x ULN | 83 | 0 | 0 | 78 | 1 | 1.3 |
|  |  |  |  |  | AST >5x to <=8x ULN | 83 | 1 | 1.2 | 78 | 0 | 0 |
|  |  |  |  |  | ALT >8x ULN | 83 | 3 | 3.6 | 78 | 0 | 0 |
|  |  |  |  |  | AST >8x ULN | 83 | 1 | 1.2 | 78 | 1 | 1.3 |
|  |  |  |  |  | Bilirubin >2x to <=3x ULN | 83 | 2 | 2.4 | 78 | 1 | 1.3 |
|  |  |  |  |  | Bilirubin >3x to <=5x ULN | 83 | 0 | 0 | 78 | 0 | 0 |
|  |  |  |  |  | Bilirubin >5x to <=8x ULN | 83 | 0 | 0 | 78 | 0 | 0 |
|  |  |  |  |  | Bilirubin >8x ULN | 83 | 0 | 0 | 78 | 0 | 0 |
| R117H | Moss (2015)^13^ | IVA | 150^2 | 24 | No LFT abnormalities reported | 34 | 0 | 0 | 35 | 0 | 0 |
| Nonsense mutation | Kerem (2014)^14^ | Ataluren | 10/10/20 mg/kg | 48 | No LFT abnormalities reported | 120 | 0 | 0 | 118 | 0 | 0 |

*****Placebo group included ivacaftor 150^2

**Abbreviations:** ALT=alanine aminotransferase; AST=aspartate aminotransferase; IVA=ivacaftor; LFT=liver function test; LUM=lumacaftor; NR=not reported; TEZ=tezacaftor; ULN=upper limit of normal.

**Appendix Figure-1. Summary of Risk of Bias for Included Studies.**


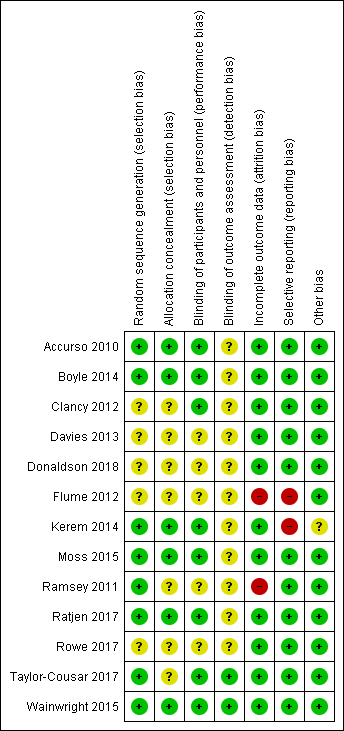


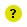
Unclear Risk of Bias


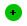
Low Risk of Bias
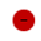
 High Risk of Bias

### **Appendix Figure-2A. Risk of Protocol-defined Pulmonary Exacerbations for CF Individuals Randomized to CFTR Modulators vs. Placebo.**


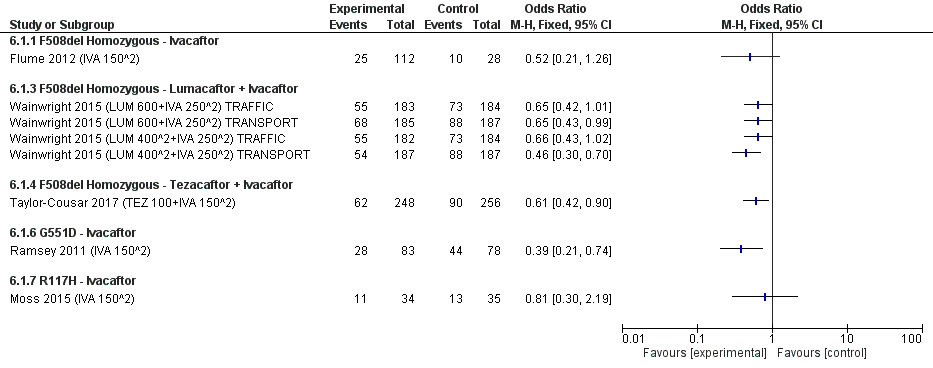


**Abbreviations:** IVA=ivacaftor; LUM=lumacaftor; TEZ=tezacaftor; ^2=twice a day

**Appendix Figure-2B. Meta-analysis of the Risk of Protocol-defined Pulmonary Exacerbations for CF Individuals Randomized to CFTR Modulators vs. Placebo.**

**
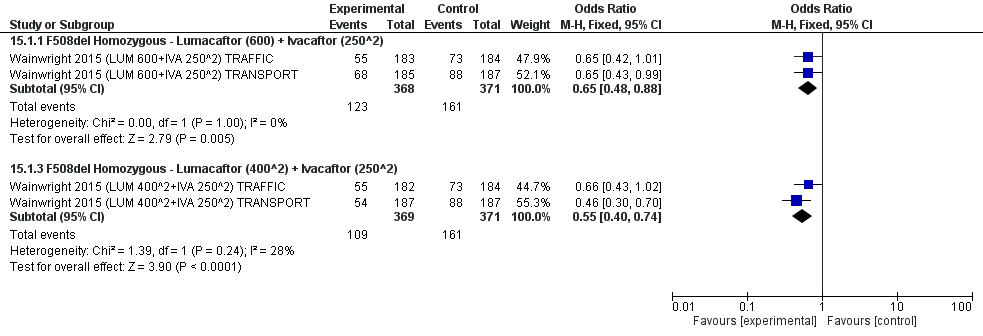
**

**Abbreviations:** IVA=ivacaftor; LUM=lumacaftor; TEZ=tezacaftor; ^2=twice a day

### **Appendix Figure-3A. Risk of Pulmonary Exacerbations requiring Hospitalization for CF Individuals Randomized to CFTR Modulators vs. Placebo.**


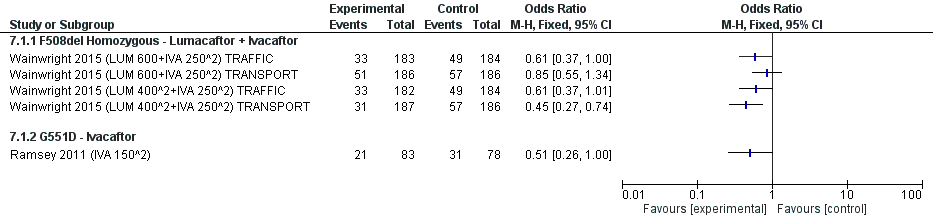


**Abbreviations:** IVA=ivacaftor; LUM=lumacaftor; ^2=twice a day

### **Appendix Figure-3B. Meta-analysis of the Risk of Pulmonary Exacerbations requiring Hospitalization for CF Individuals Randomized to CFTR Modulators vs. Placebo.**

**
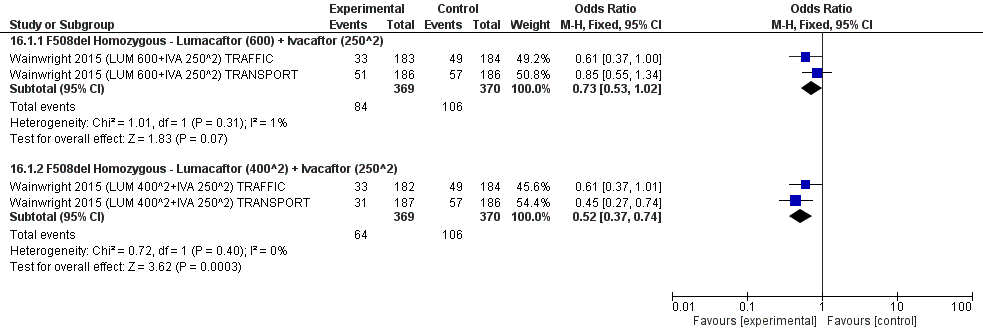
 Abbreviations:** IVA=ivacaftor; LUM=lumacaftor; ^2=twice a day

### **Appendix Figure-4A. Absolute Difference in CFQ-R Resp Domain Scores for CF Individuals Randomized to CFTR Modulators vs. Placebo.**

**
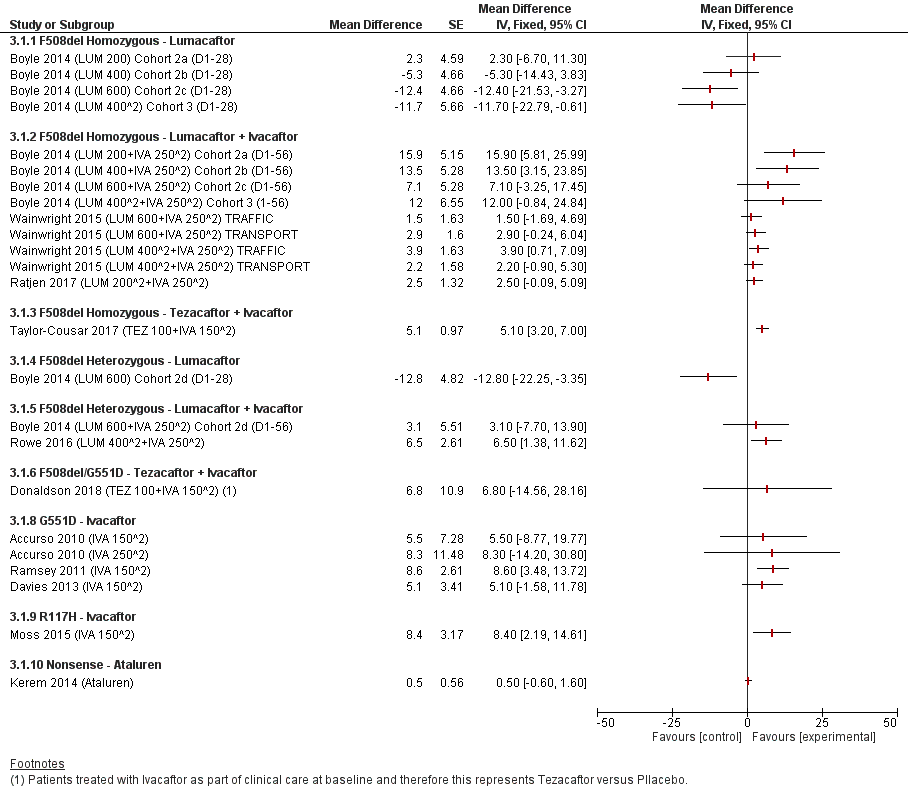
**

**Footnote:** (1) Individuals received IVA at baseline as part of routine clinical care and therefore the control group received IVA + Placebo.

**Abbreviations:** D1-28=day 1 to day 28; D1-56=day 1 to day 56; IVA=ivacaftor; LUM=lumacaftor; TEZ=tezacaftor; ^2=twice a day

### **Appendix Figure-4B. Meta-analysis of the Absolute Difference in CFQ-R Resp Domain Scores for CF Individuals Randomized to CFTR Modulators vs. Placebo.**

**
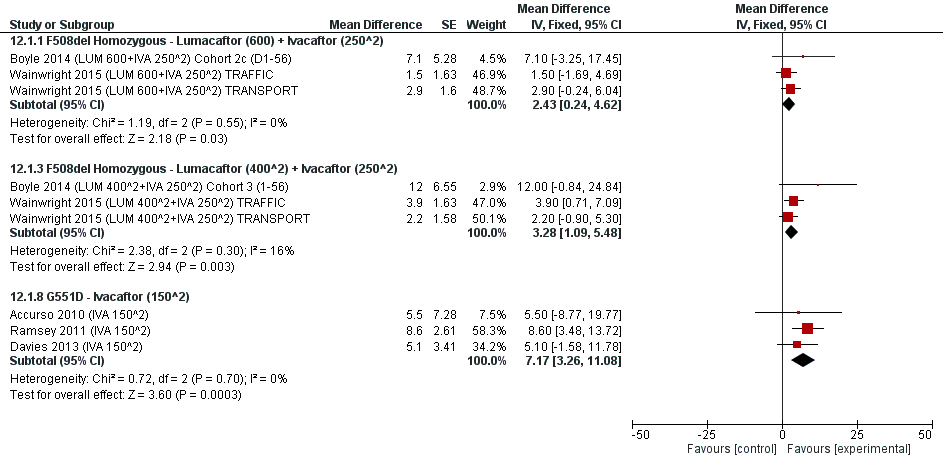
**

**Abbreviations:** D1-56=day 1 to day 56; IVA=ivacaftor; LUM=lumacaftor; TEZ=tezacaftor; ^2=twice a day

**Appendix Figure-5A. Absolute Difference in Weight (kg) Changes for CF Individuals Randomized to CFTR Modulators vs. Placebo.**

**
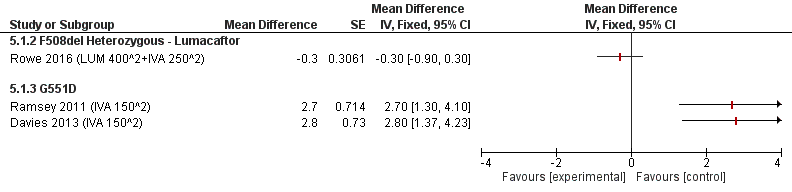
**

**Abbreviations:** IVA=ivacaftor; LUM=lumacaftor; ^2=twice a day

### **Appendix Figure-5B. Meta-analysis of the Absolute Difference in Weight (kg) Changes for CF Individuals Randomized to CFTR Modulators vs. Placebo.**

**
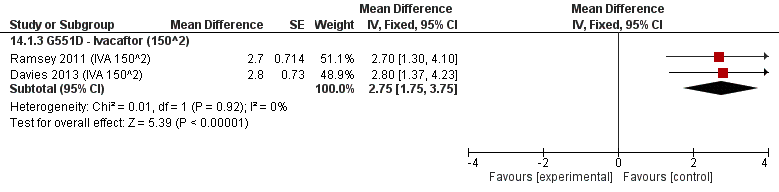
**

**Abbreviations:** IVA=ivacaftor; ^2=twice a day

**Appendix Figure-6A. Absolute Difference in BMI (kg/m^2^) Changes for CF Individuals Randomized to CFTR Modulators vs. Placebo.**


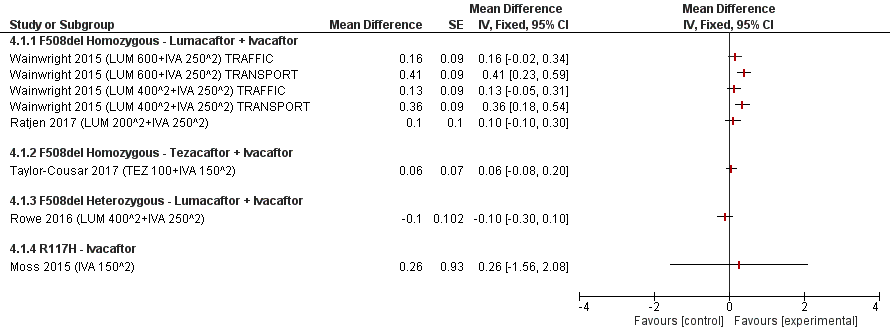


**Abbreviations:** IVA=ivacaftor; LUM=lumacaftor; TEZ=tezacaftor; ^2=twice a day

### **Appendix Figure-6B. Meta-analysis of the Absolute Difference in BMI (kg/m^2^) Changes for CF Individuals Randomized to CFTR Modulators vs. Placebo.**

**
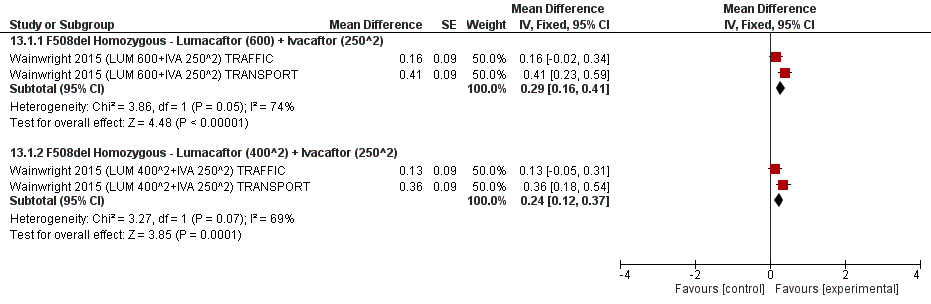
**

**Abbreviations:** IVA=ivacaftor; LUM=lumacaftor; ^2=twice a day

**Appendix Figure-7. Adverse Events: Risk of Cough for CF Individuals Randomized to CFTR Modulators vs. Placebo.**

**
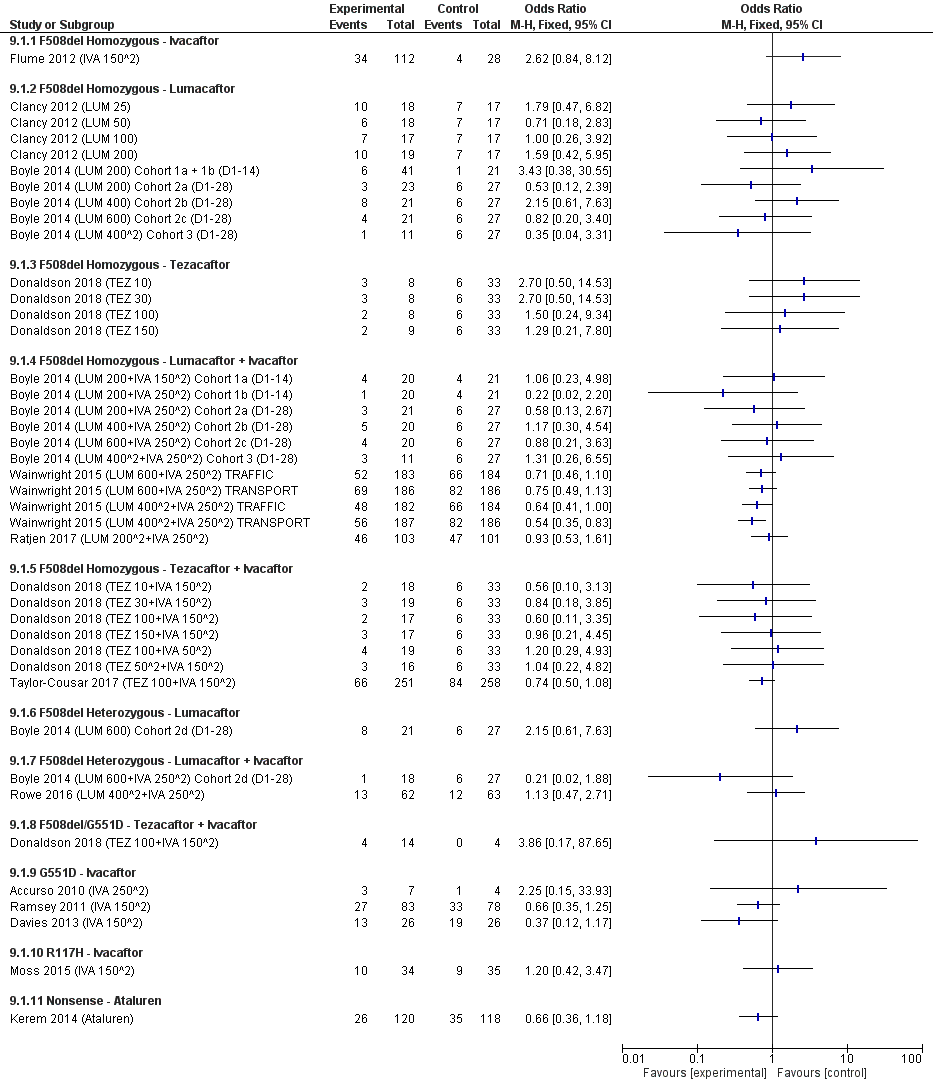
**

**Footnote:** F508del/G551D Individuals received IVA at baseline as part of routine clinical care and therefore the control group received IVA + Placebo.

**Abbreviations:** D1-14=day 1 to day 14; D1-28=day 1 to day 28; IVA=ivacaftor; LUM=lumacaftor; TEZ=tezacaftor; ^2=twice a day

**Appendix Figure-8. Adverse Events: Risk of Productive Cough for CF Individuals Randomized to CFTR Modulators vs. Placebo.**

**
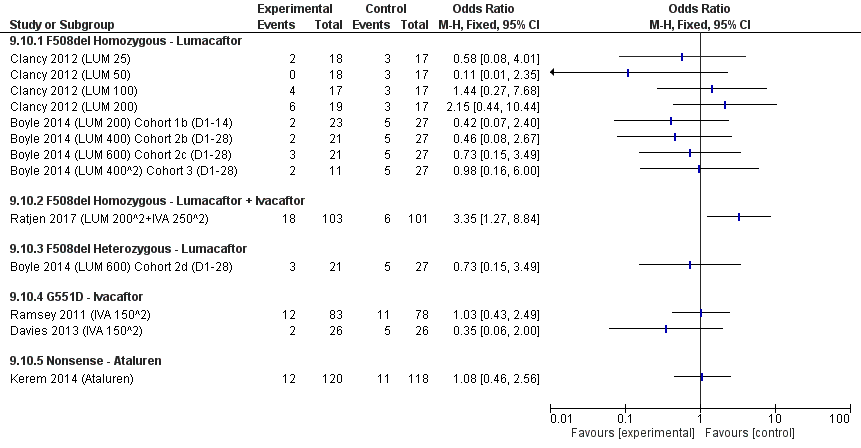
**

**Abbreviations:** D1-14=day 1 to day 14; D1-28=day 1 to day 28; IVA=ivacaftor; LUM=lumacaftor; ^2=twice a day

**Appendix Figure-9. Adverse Events: Risk of Sputum Production for CF Individuals Randomized to CFTR Modulators vs. Placebo.**

**
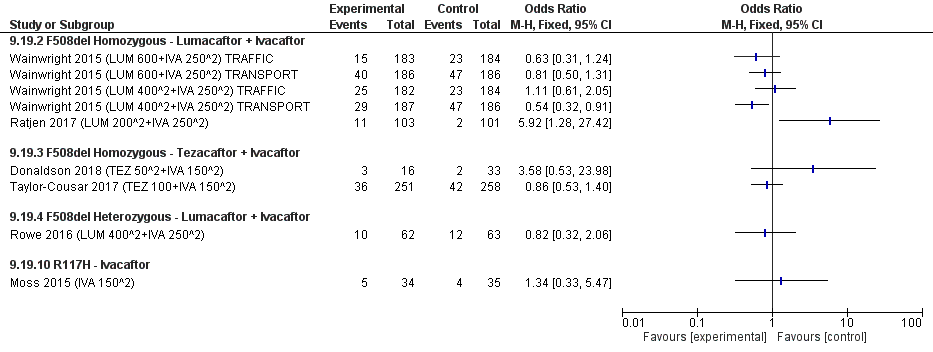
**

**Abbreviations:** IVA=ivacaftor; LUM=lumacaftor; TEZ=tezacaftor; ^2=twice a day

**Appendix Figure-10. Adverse Events: Risk of Hemoptysis for CF Individuals Randomized to CFTR Modulators vs. Placebo.**

**
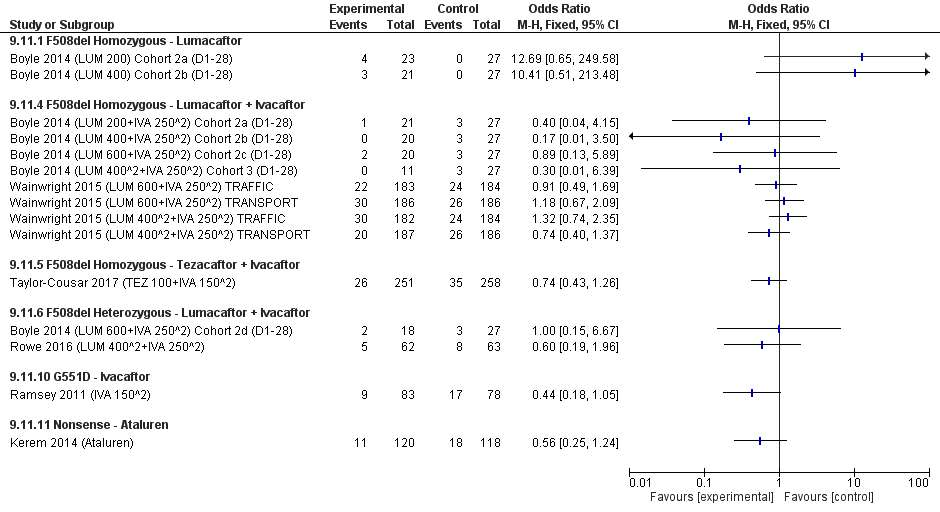
**

**Abbreviations:** D1-28=day 1 to day 28; IVA=ivacaftor; LUM=lumacaftor; TEZ=tezacaftor; ^2=twice a day

**Appendix Figure-11. Adverse Events: Risk of Dyspnea for CF Individuals Randomized to CFTR Modulators vs. Placebo.**

**
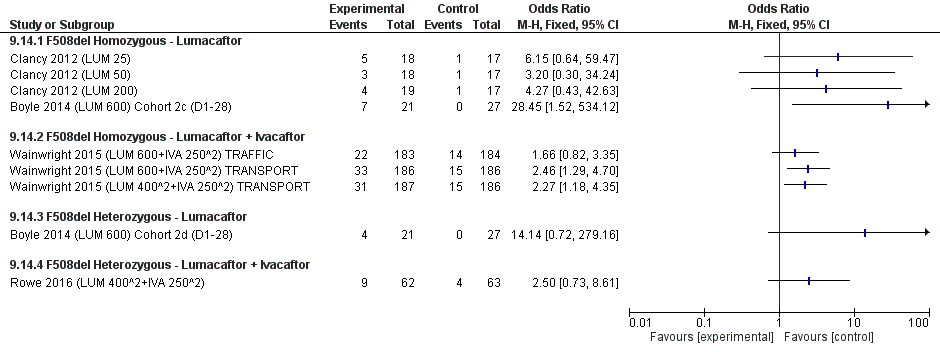
**

**Abbreviations:** D1-28=day 1 to day 28; IVA=ivacaftor; LUM=lumacaftor; ^2=twice a day

**Appendix Figure-12. Adverse Events: Risk of Wheezing for CF Individuals Randomized to CFTR Modulators vs. Placebo.**

**
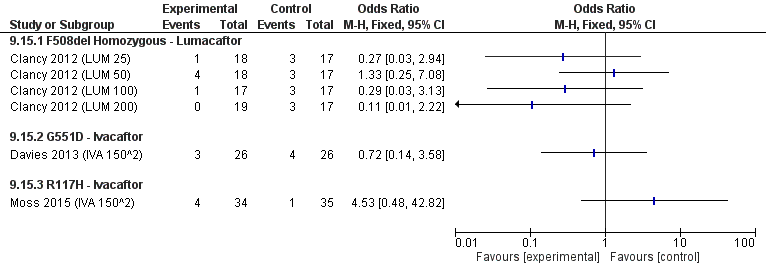
**

**Footnote:** (1) Individuals received IVA at baseline as part of routine clinical care and therefore the control group received IVA + Placebo.

**Abbreviations:** D1-14=day 1 to day 14; D1-21=day 1 to day 21; D1-28=day 1 to day 28; D1-56=day 1 to day 56; IVA=ivacaftor; LUM=lumacaftor; TEZ=tezacaftor; ^2=twice a day

**Appendix Figure-13. Adverse Events: Risk of Abnormal Respiration for CF Individuals Randomized to CFTR Modulators vs. Placebo.**

**
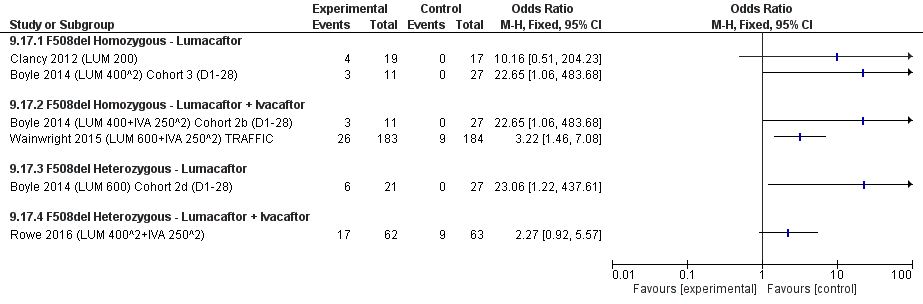
**

**Abbreviations:** D1-28=day 1 to day 28; IVA=ivacaftor; LUM=lumacaftor; ^2=twice a day

**Appendix Figure-14. Adverse Events: Risk of Reduced Pulmonary Function for CF Individuals Randomized to CFTR Modulators vs. Placebo.**

**
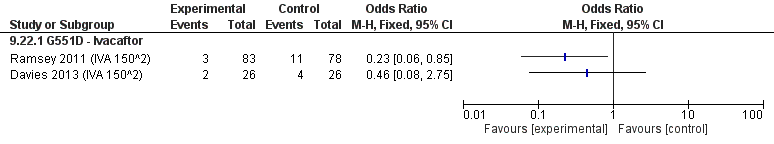
**

**Abbreviations:** IVA=ivacaftor; ^2=twice a day

**Appendix Figure-15. Adverse Events: Risk of Rales for CF Individuals Randomized to CFTR Modulators vs. Placebo.**

**
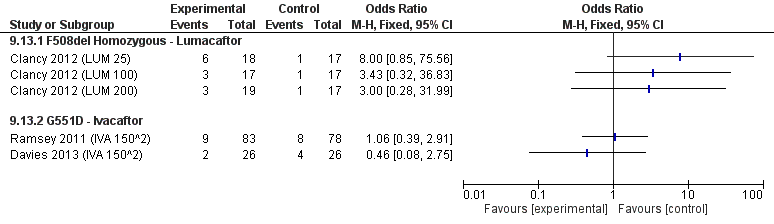
**

**Abbreviations:** IVA=ivacaftor; LUM=lumacaftor; ^2=twice a day

**Appendix Figure-16. Adverse Events: Pulmonary Exacerbation Risk for CF Individuals Randomized to CFTR Modulators vs. Placebo.**

**
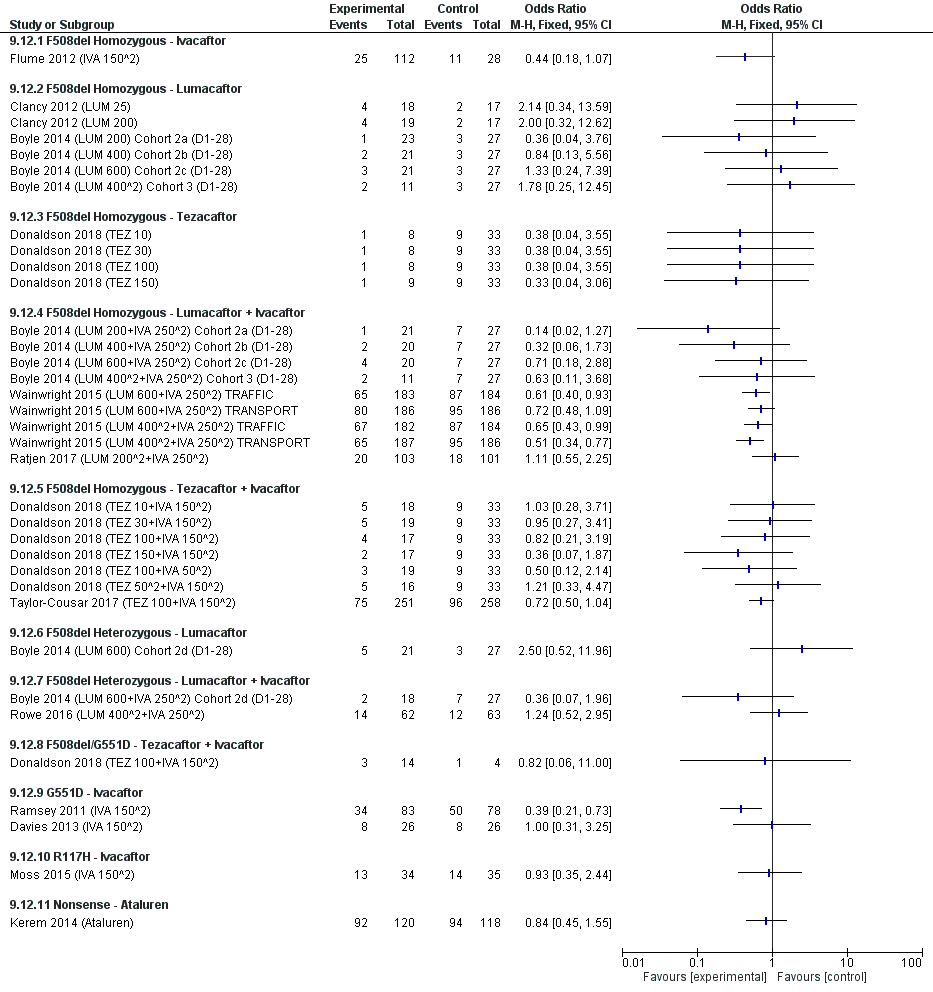
**

**Footnote:** (1) Individuals received IVA at baseline as part of routine clinical care and therefore the control group received IVA + Placebo.

**Abbreviations:** D1-28=day 1 to day 28; IVA=ivacaftor; LUM=lumacaftor; TEZ=tezacaftor; ^2=twice a day

**Appendix Figure-17. Adverse Events: Risk of Upper Respiratory Tract Infection for CF Individuals Randomized to CFTR Modulators vs. Placebo.**

**
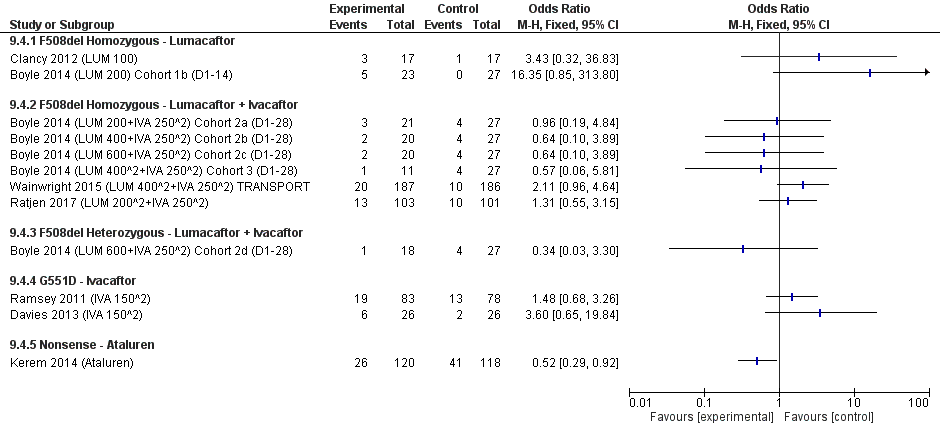
**

**Abbreviations:** D1-14=day 1 to day 14; D1-28=day 1 to day 28; IVA=ivacaftor; LUM=lumacaftor; ^2=twice a day

**Appendix Figure-18. Adverse Events: Risk of Sinus Congestion/Sinusitis for CF Individuals Randomized to CFTR Modulators vs. Placebo.**

**
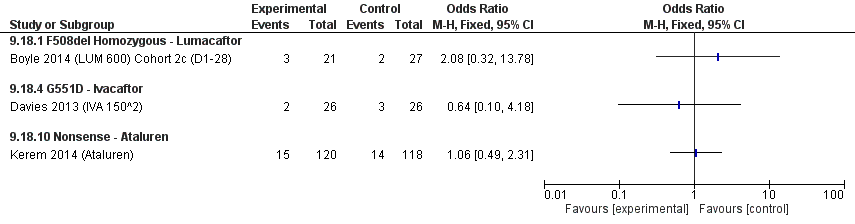
**

**Abbreviations:** IVA=ivacaftor; LUM=lumacaftor; ^2=twice a day

**Appendix Figure-19. Adverse Events: Risk of Nasal Congestion for CF Individuals Randomized to CFTR Modulators vs. Placebo.**

**
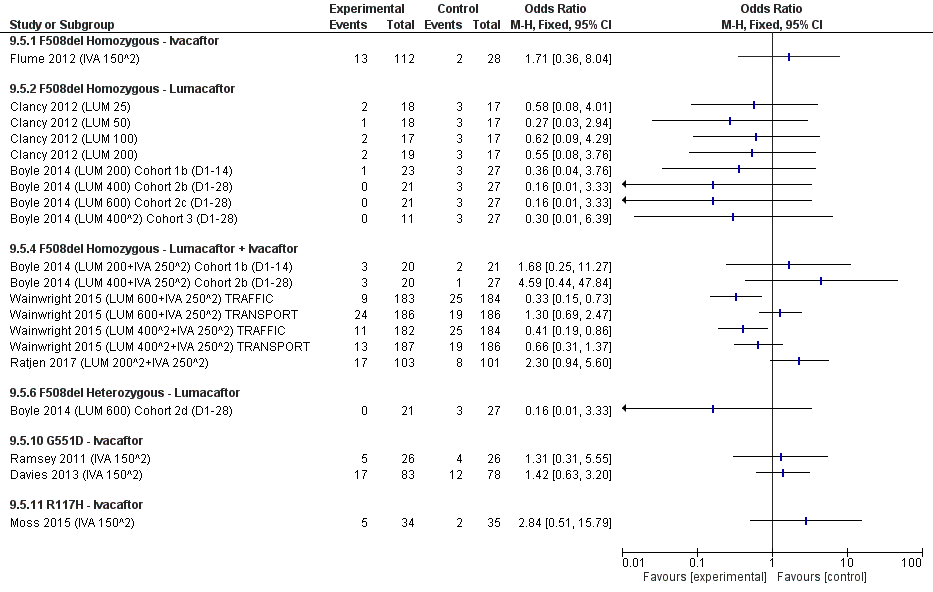
**

**Abbreviations:** D1-14=day 1 to day 14; D1-28=day 1 to day 28; IVA=ivacaftor; LUM=lumacaftor; ^2=twice a day

**Appendix Figure-20. Adverse Events: Risk of Rhinitis for CF Individuals Randomized to CFTR Modulators vs. Placebo.**

**
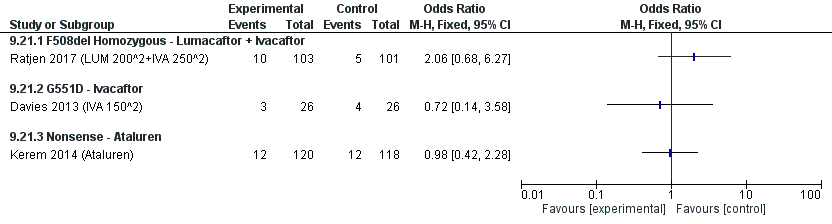
**

**Abbreviations:** IVA=ivacaftor; LUM=lumacaftor; ^2=twice a day

**Appendix Figure-21. Adverse Events: Risk of Nasopharyngitis for Patients CF Individuals Randomized to CFTR Modulators vs. Placebo.**

**
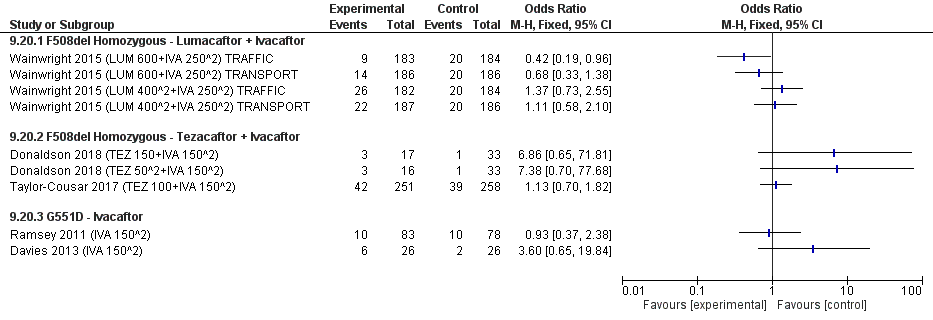
**

.

**Abbreviations:** IVA=ivacaftor; LUM=lumacaftor; TEZ=tezacaftor; ^2=twice a day

**Appendix Figure-22. Adverse Events: Risk of Oropharyngeal Pain for CF Individuals Randomized to CFTR Modulators vs. Placebo.**

**
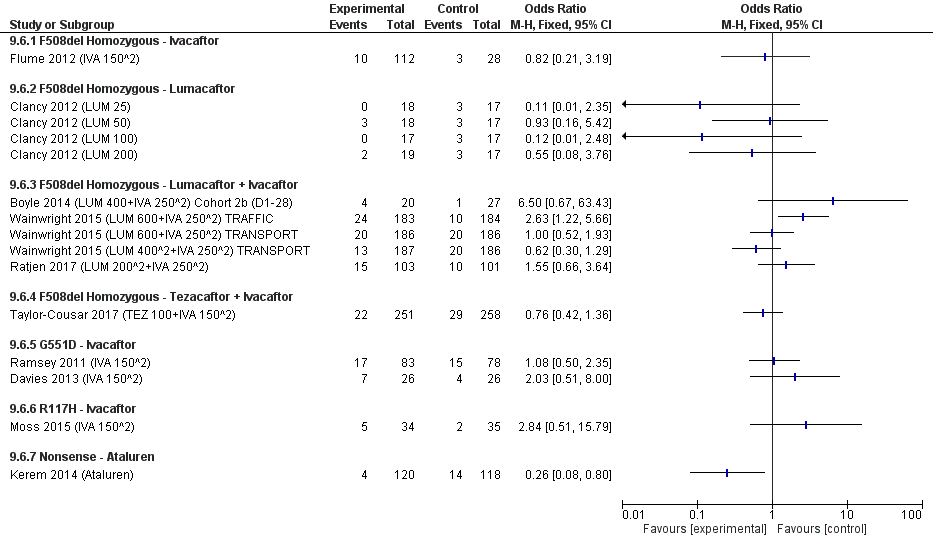
**

**Abbreviations:** D1-28=day 1 to day 28; D1-56=day 1 to day 56; IVA=ivacaftor; LUM=lumacaftor; TEZ=tezacaftor; ^2=twice a day

**Appendix Figure-23. Adverse Events: Risk of Fever for CF Individuals Randomized to CFTR Modulators vs. Placebo.**

**
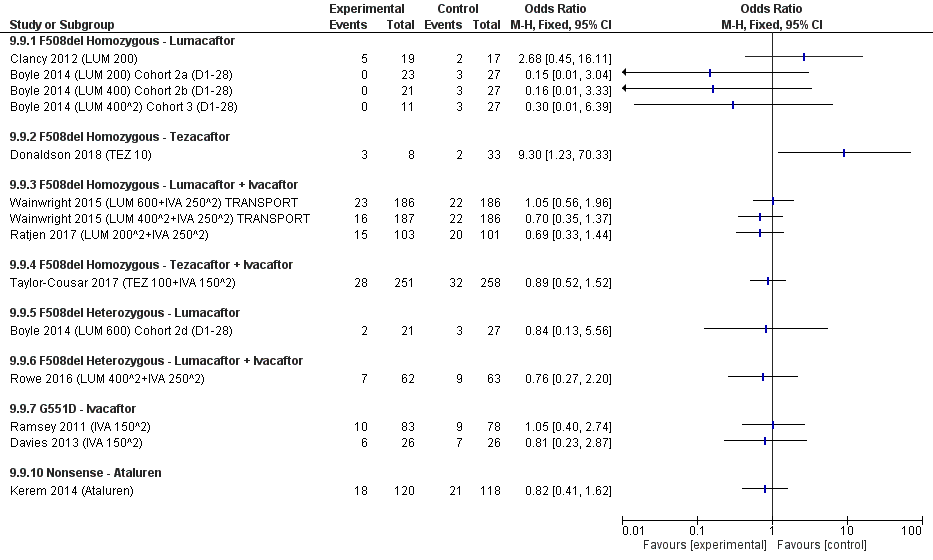
**

**Abbreviations:** D1-28=day 1 to day 28; IVA=ivacaftor; LUM=lumacaftor; TEZ=tezacaftor; ^2=twice a day

**Appendix Figure-24. Adverse Events: Risk of Fatigue for CF Individuals Randomized to CFTR Modulators vs. Placebo.**

**
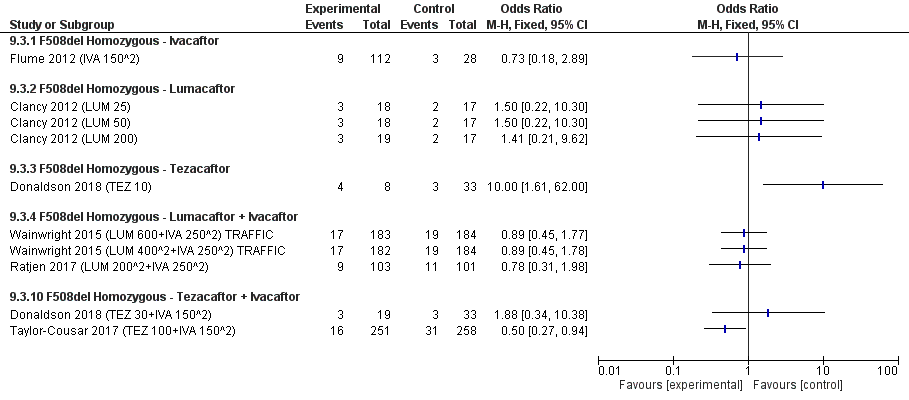
**

**Abbreviations:** IVA=ivacaftor; LUM=lumacaftor; TEZ=tezacaftor; ^2=twice a day

**Appendix Figure-25. Adverse Events: Risk of Nausea for CF Individuals Randomized to CFTR Modulators vs. Placebo.**

**
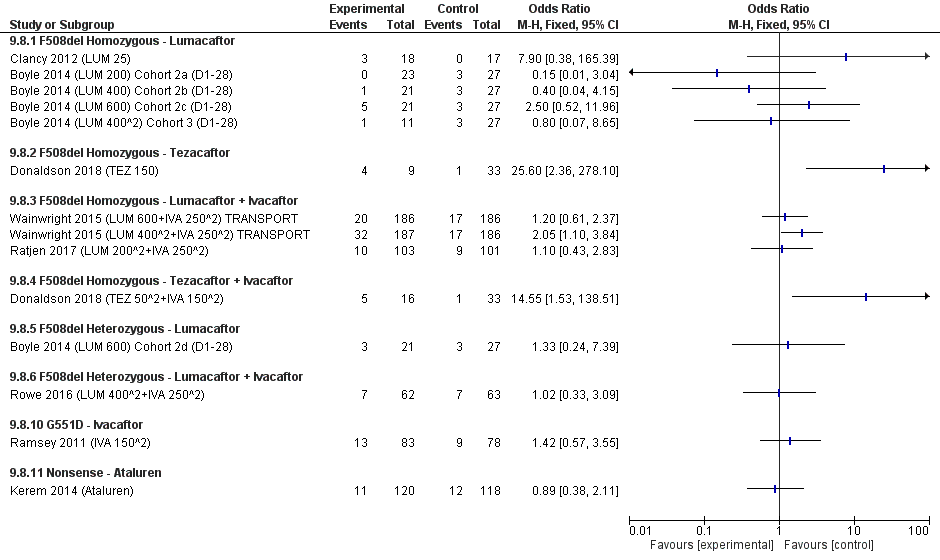
**

**Abbreviations:** D1-28=day 1 to day 28; IVA=ivacaftor; LUM=lumacaftor; TEZ=tezacaftor; ^2=twice a day

**Appendix Figure-26. Adverse Events: Risk of Vomiting for CF Individuals Randomized to CFTR Modulators vs. Placebo.**

**
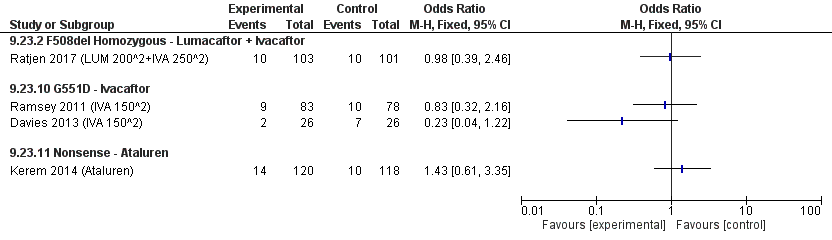
**

**Abbreviations:** IVA=ivacaftor; LUM=lumacaftor; ^2=twice a day

**Appendix Figure-27. Adverse Events: Risk of Abdominal Pain for CF Individuals Randomized to CFTR Modulators vs. Placebo.**

**
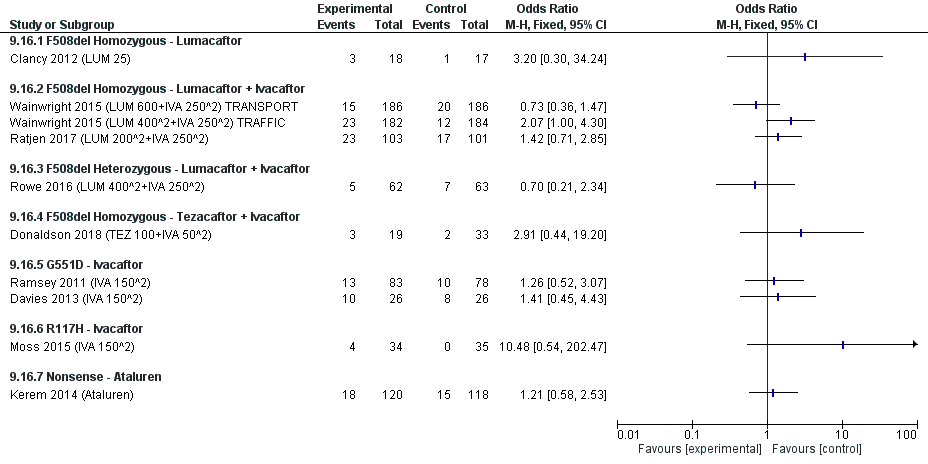
**

**Abbreviations:** IVA=ivacaftor; LUM=lumacaftor; TEZ=tezacaftor; ^2=twice a day

**Appendix Figure-28. Adverse Events: Risk of Diarrhea for CF Individuals Randomized to CFTR Modulators vs. Placebo.**

**
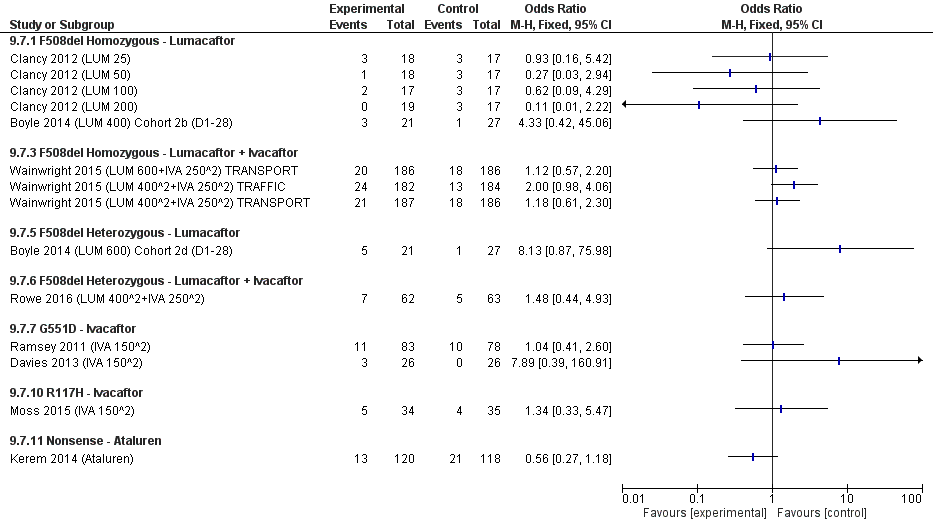
**

**Abbreviations:** D1-28=day 1 to day 28; IVA=ivacaftor; LUM=lumacaftor; ^2=twice a day

**Appendix Figure-29. Adverse Events: Risk of Headache for CF Individuals Randomized to CFTR Modulators vs. Placebo.**

**
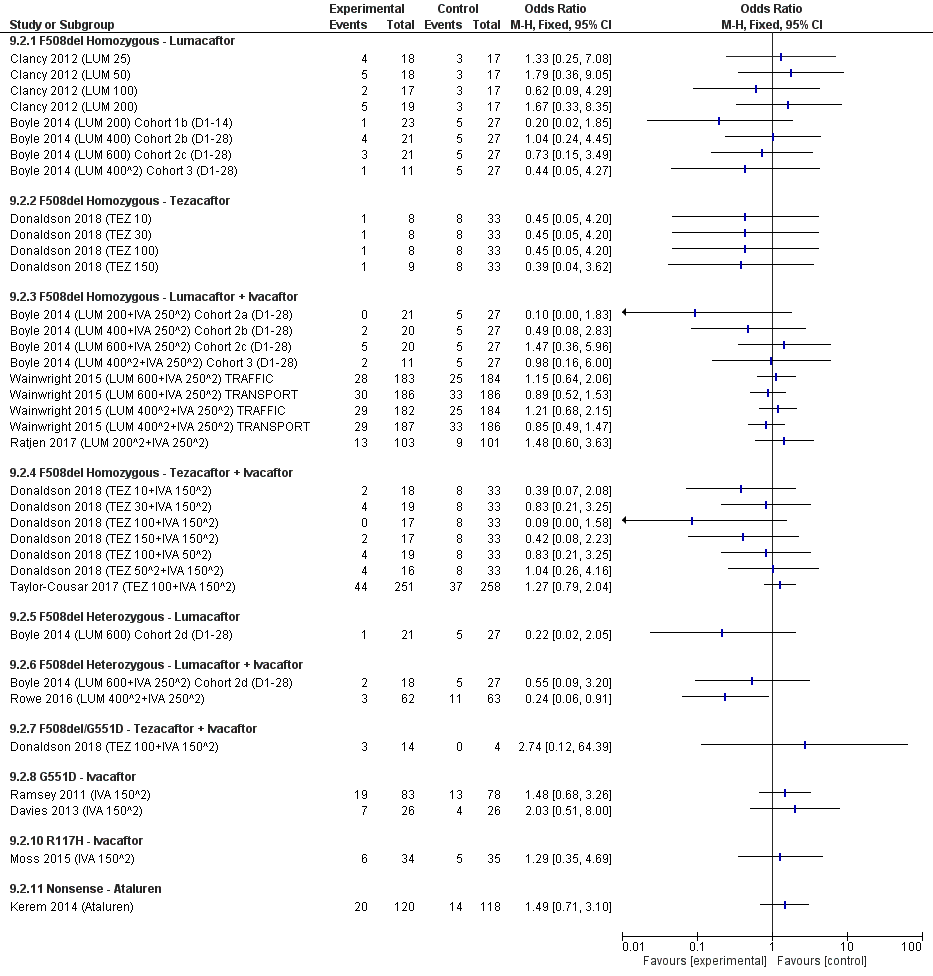
**

**Abbreviations:** D1-14=day 1 to day 14; D1-28=day 1 to day 28; IVA=ivacaftor; LUM=lumacaftor; TEZ=tezacaftor; ^2=twice a day

**Appendix Figure-30. Adverse Events: Risk of Rash for CF Individuals Randomized to CFTR Modulators vs. Placebo.**

**
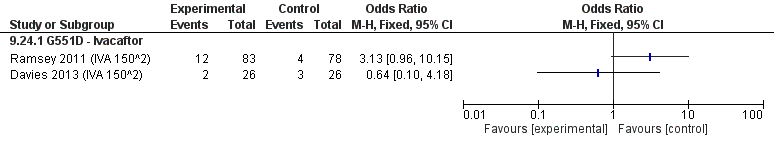
**

**Abbreviations:** IVA=ivacaftor; ^2=twice a day

**Study Review Protocol**

**Proposed Title:** Systematic Review of the effects of CFTR modulators on clinically important outcomes among individuals with cystic fibrosis

**Objective:** To evaluate the effects of CFTR modulators on lung function, pulmonary exacerbations, nutritional status, respiratory symptoms, and safety in children and adults with cystic fibrosis.

**Clinically Important Outcomes:**

**Primary**

1. Lung function (pulmonary function tests measured in litres or percent predicted adjusted for age, sex and height)
   1. Forced expiratory volume in one second (FEV_1_)
      1. Primary: Absolute change from baseline in FEV1 % predicted
      2. Secondary: Relative change from baseline in FEV1 % predicted

**Secondary**

1. Pulmonary exacerbations
   1. Protocol-defined exacerbations (as defined by the clinical trial protocol)
   2. Events defined by hospitalization
2. Respiratory symptoms (i.e. Cystic Fibrosis Questionnaire-Revised)
   1. CFQ-R respiratory domain
3. Nutritional status
   1. Weight change from baseline in kg
   2. BMI change from baseline in kg/m2
4. Adverse events
   1. Events leading to discontinuation of study drug
   2. Adverse events with frequency >= 10% in either placebo or treatment groups
5. Consider the following pre-specified sub-group analyses to see if the treatment effects differ based on: age (paediatrics vs. adults), disease severity based on FEV1 (mild vs. mod-severe).

**CFTR Modulators of Interest:**

1. Potentiators
2. Correctors
3. Read-through agents:
   1. Aminoglycoside antibiotics and derivatives (gentamicin, tobramycin)
   2. Non-aminoglycoside: NB124, Ataluren
4. Any others identified
5. Combination therapies

**Selection Criteria:** Randomized controlled trials (parallel design) comparing CFTR modulators to placebo or other treatment, in people with cystic fibrosis. Study participants of any level of disease severity and confirmed cystic fibrosis diagnosis from positive sweat, genotype or clinical phenotype will be included.

**Search Strategy:** A search of multiple literature databases will be conducted to review randomized controlled trials published between January 1, 2005 to March 31, 2018. Literature databases will include MEDLINE, EMBASE, ACP Journal Club, Cochrane Central Register for Controlled Trials (CENTRAL), Cochrane Database of Systematic Reviews (CDSR), Cochrane Methodology Register (CMR), Database of Abstracts of Reviews of Effects (DARE), Health Technology Assessment (HTA), and NHS Economic Evaluation Database (NHSEED). For comprehensiveness, clinical trial registries such as the European Medicines Agency, U.S. National Institute of Health, and the World Health Organization records will be accessed and screened. The following key phrases will be included to maximize sensitivity for detecting therapeutic trials in CF: (“cystic fibrosis” OR “CFTR”) AND (“drug therapy” OR “clinical trial”).

**References**

1. Higgins JP, Green S. Cochrane Handbook for Systematic Reviews of Interventions Version 5.1.0 [updated March 2011]. The Cochrane Collaboration, 2011. Available from [http://handbook.cochrane.org/](http://handbook.cochrane.org).

2. Flume PA, Liou TG, Borowitz DS, et al. Ivacaftor in subjects with cystic fibrosis who are homozygous for the F508del-CFTR mutation. *Chest* 2012; **142**(3): 718-24.

3. Clancy JP, Rowe SM, Accurso FJ, et al. Results of a phase IIa study of VX-809, an investigational CFTR corrector compound, in subjects with cystic fibrosis homozygous for the F508del-CFTR mutation. *Thorax* 2012; **67**(1): 12-8.

4. Boyle MP, Bell SC, Konstan MW, et al. A CFTR corrector (lumacaftor) and a CFTR potentiator (ivacaftor) for treatment of patients with cystic fibrosis who have a phe508del CFTR mutation: a phase 2 randomised controlled trial. *Lancet Respir Med* 2014; **2**(7): 527-38.

5. Donaldson SH, Pilewski JM, Griese M, et al. Tezacaftor/Ivacaftor in Subjects with Cystic Fibrosis and F508del/F508del-CFTR or F508del/G551D-CFTR. *Am J Respir Crit Care Med* 2018; **197**(2): 214-24.

6. Wainwright CE, Elborn JS, Ramsey BW, et al. Lumacaftor-Ivacaftor in Patients with Cystic Fibrosis Homozygous for Phe508del CFTR. *N Engl J Med* 2015; **373**(3): 220-31.

7. Ratjen F, Hug C, Marigowda G, et al. Efficacy and safety of lumacaftor and ivacaftor in patients aged 6-11 years with cystic fibrosis homozygous for F508del-CFTR: a randomised, placebo-controlled phase 3 trial. *Lancet Respir Med* 2017; **5**(7): 557-67.

8. Taylor-Cousar JL, Munck A, McKone EF, et al. Tezacaftor-Ivacaftor in Patients with Cystic Fibrosis Homozygous for Phe508del. *N Engl J Med* 2017; **377**(21): 2013-23.

9. Rowe SM, McColley SA, Rietschel E, et al. Lumacaftor/Ivacaftor Treatment of Patients with Cystic Fibrosis Heterozygous for F508del-CFTR. *Ann Am Thorac Soc* 2017; **14**(2): 213-9.

10. Accurso FJ, Rowe SM, Clancy JP, et al. Effect of VX-770 in persons with cystic fibrosis and the G551D-CFTR mutation. *N Engl J Med* 2010; **363**(21): 1991-2003.

11. Ramsey BW, Davies J, McElvaney NG, et al. A CFTR potentiator in patients with cystic fibrosis and the G551D mutation. *N Engl J Med* 2011; **365**(18): 1663-72.

12. Davies JC, Wainwright CE, Canny GJ, et al. Efficacy and safety of ivacaftor in patients aged 6 to 11 years with cystic fibrosis with a G551D mutation. *Am J Respir Crit Care Med* 2013; **187**(11): 1219-25.

13. Moss RB, Flume PA, Elborn JS, et al. Efficacy and safety of ivacaftor in patients with cystic fibrosis who have an Arg117His-CFTR mutation: a double-blind, randomised controlled trial. *Lancet Respir Med* 2015; **3**(7): 524-33.

14. Kerem E, Konstan MW, De Boeck K, et al. Ataluren for the treatment of nonsense-mutation cystic fibrosis: a randomised, double-blind, placebo-controlled phase 3 trial. *Lancet Respir Med* 2014; **2**(7): 539-47.
